# Supplementary material for: Predicting species assemblages at wildlife crossing structures using multivariate regression of principal coordinates
Source: PLoS One. 2025 Oct 24;20(10):e0335193. doi: 10.1371/journal.pone.0335193 (PMC12551880; doi:10.1371/journal.pone.0335193)
Supplement: S1 Appendix — (DOCX) [file pone.0335193.s001.docx]

**Supporting Information**

Predicting species assemblages at wildlife crossing structures using multivariate regression of principal coordinates

**Appendix S1: Description of the characteristics used to predict the mammal community composition of each wildlife crossing structure (WCS).**

Table S1.1: General description, spatial, and temporal characteristics of each wildlife crossing structure (WCS) built on three highways in South Texas (Farm-to-Market [FM] 106, FM 1847, and State Highway [SH] 100) for ocelots. Description of each WCS on each of the three highways (Farm-to-Market [FM] 106, FM 1847, and State Highway [SH] 100).

| Highway | WCS | Opening Date | Monitoring End | WCS Type | Latitude | Longitude |
| --- | --- | --- | --- | --- | --- | --- |
| FM 106 | WCS1 | Jul 2019 | Dec 2020 | Culvert | 26.204177 | -97.420034 |
|  | WCS2 | Jul 2019 | Dec 2020 | Culvert | 26.199451 | -97.413166 |
|  | WCS3 | Jul 2019 | Dec 2020 | Culvert | 26.194090 | -97.384234 |
|  | WCS4 | Jul 2019 | Dec 2020 | Culvert | 26.192262 | -97.363579 |
|  | WCS5 | Jul 2019 | Dec 2020 | Culvert | 26.192181 | -97.362792 |
|  | WCS6 | Jul 2019 | Dec 2020 | Culvert | 26.152224 | -97.351108 |
|  | WCS7 | Jul 2019 | Dec 2020 | Culvert | 26.144417 | -97.350723 |
|  | WCS8 | Jul 2019 | Dec 2020 | Culvert | 26.134307 | -97.350322 |
| FM 1847 | WCS1 | Jan 2022 | Aug 2023 | Culvert | 26.117107 | -97.472247 |
|  | WCS2 | Jan 2022 | Aug 2023 | Bridge | 26.147466 | -97.469561 |
|  | WCS3 | Mar 2022 | Aug 2023 | Culvert | 26.152291 | -97.469138 |
|  | WCS4 | Mar 2022 | Aug 2023 | Culvert | 26.165604 | -97.467964 |
|  | WCS5 | Mar 2022 | Aug 2023 | Culvert | 26.166758 | -97.467868 |
| SH 100 | WCS1 | Oct 2017 | May 2019 | Culvert | 26.0671823 | -97.412810 |
|  | WCS2 | Oct 2017 | May 2019 | Culvert | 26.0658958 | -97.394909 |
|  | WCS3 | Dec 2017 | May 2019 | Bridge | 26.0713153 | -97.376622 |
|  | WCS3A^1^ | Sep 2016 | May 2019 | Culvert | 26.0754247 | -97.372506 |
|  | WCS4 | Dec 2017 | May 2019 | Culvert | 26.0917170 | -97.329810 |

^1^This was a pre-existing WCS and only received minor modifications and remained open during construction of the other WCSs.

Table S1.2: Structural characteristics of each wildlife crossing structure (WCS) built on three highways in South Texas (Farm-to-Market [FM] 106, FM 1847, and State Highway [SH] 100) for ocelots.

| Highway | WCS | Fencing Length (m) | Catwalk | Substrate | Openness Ratio (m)^5^ |
| --- | --- | --- | --- | --- | --- |
| FM 106 | WCS1 | 1223.2 | None | Concrete | 0.144 |
|  | WCS2 | 807.1 | None | Concrete | 0.244 |
|  | WCS3 | 838.2 | Concrete Step | Dirt | 0.122 |
|  | WCS4 | 286.2^1^ | None | Concrete | 0.127 |
|  | WCS5 | 286.2^1^ | None | Concrete | 0.152 |
|  | WCS6 | 146 | Concrete step | Dirt | 0.149 |
|  | WCS7 | 274.9 | Concrete step | Dirt | 0.122 |
|  | WCS8 | 420.9 | None | Concrete | 0.144 |
| FM 1847 | WCS1 | 114.6^2^ | Concrete step | Concrete | 0.107 |
|  | WCS2 | 181.8^2^ | Dirt bench | Dirt | 3.211 |
|  | WCS3 | 181.4^2^ | Concrete step | Concrete | 0.178 |
|  | WCS4 | 202.4^3^ | Concrete step | Concrete | 0.181 |
|  | WCS5 | 202.4^3^ | Concrete step | Concrete | 0.155 |
| SH 100 | WCS1 | 11104.5^4^ | Concrete step | Water | 0.133 |
|  | WCS2 | 11104.5^4^ | Concrete step | Water | 0.119 |
|  | WCS3 | 11104.5^4^ | None | Dirt | 0.535 |
|  | WCS3A | 11104.5^4^ | None | Dirt | 0.064 |
|  | WCS4 | 11104.5^4^ | None | Concrete | 0.191 |

^1^Fencing was shared between WCS4 and WCS5 on FM 106.

^2^Fencing lengths differed on the east and west sides of the highway. The lengths were averaged prior to analysis.

^3^Fencing was shared between WCS4 and WCS5 on FM 1847.

^4^Fencing was shared between all WCS on SH 100.

^5^Openness ratio is calculated as: $(\frac{height \times width}{length})$.

Table S1.3: Environmental characteristics of each wildlife crossing structure (WCS) built on three highways in South Texas (Farm-to-Market [FM] 106, FM 1847, and State Highway [SH] 100) for ocelots.

| Highway | WCS | Prop. Natural | Prop. Woody | Prop. Water | Precip. (mm)^1^ |
| --- | --- | --- | --- | --- | --- |
| FM 106 | WCS1 | 0.987 | 0.323 | 0.002 | 37.2 ± 41.4 |
|  | WCS2 | 0.987 | 0.304 | 0.003 | 37.2 ± 41.4 |
|  | WCS3 | 0.410 | 0.061 | 0.001 | 37.2 ± 41.4 |
|  | WCS4 | 0.231 | 0.061 | 0.054 | 37.2 ± 41.4 |
|  | WCS5 | 0.225 | 0.059 | 0.052 | 37.2 ± 41.4 |
|  | WCS6 | 0.735 | 0.341 | 0.016 | 37.2 ± 41.4 |
|  | WCS7 | 0.891 | 0.310 | 0.007 | 37.2 ± 41.4 |
|  | WCS8 | 0.971 | 0.114 | 0.009 | 37.2 ± 41.4 |
| FM 1847 | WCS1 | 0.729 | 0.218 | 0.006 | 41.5 ± 39.3 |
|  | WCS2 | 0.790 | 0.281 | 0.005 | 41.5 ± 39.3 |
|  | WCS3 | 0.678 | 0.195 | 0.010 | 39 ± 40.7 |
|  | WCS4 | 0.878 | 0.116 | 0.124 | 39 ± 40.7 |
|  | WCS5 | 0.900 | 0.113 | 0.128 | 39 ± 40.7 |
| SH 100 | WCS1 | 0.831 | 0.050 | 0.001 | 51.2 ± 50 |
|  | WCS2 | 0.970 | 0.163 | 0.011 | 51.2 ± 50 |
|  | WCS3 | 0.979 | 0.189 | 0.001 | 51.1 ± 52.7 |
|  | WCS3A | 0.980 | 0.184 | 0.001 | 44.9 ± 44.4 |
|  | WCS4 | 0.981 | 0.007 | 0.001 | 51.1 ± 52.7 |

^1^Precipitation was computed from a single weather station in the region. The mean and standard deviation of precipitation during the time of monitoring is provided.

Table S1.4: Anthropogenic characteristics of each wildlife crossing structure (WCS) built on three highways in South Texas (Farm-to-Market [FM] 106, FM 1847, and State Highway [SH] 100) for ocelots.

| Highway | WCS | Speed Limit^1^ | Building area | Vehicle traffic^2^ | Human activity^3^ | Domestic activity^3^ | Livestock activity^3^ |
| --- | --- | --- | --- | --- | --- | --- | --- |
| FM 106 | WCS1 | 88, 97 | 0.0002 | 852, 743 | 7.61 ± 11.5 | 0.167 ± 0.514 | 0 |
|  | WCS2 | 88, 97 | 0.0000 | 852, 743 | 6.39 ± 10.5 | 0.278 ± 0.752 | 0 |
|  | WCS3 | 88, 97 | 0.0015 | 852, 743 | 2.56 ± 2.71 | 1.39 ± 1.82 | 0 |
|  | WCS4 | 88, 97 | 0.0002 | 852, 743 | 4.83 ± 6.09 | 0.333 ± 0.686 | 0 |
|  | WCS5 | 88, 97 | 0.0002 | 852, 743 | 6.67 ± 11.6 | 0.944 ± 2.46 | 0 |
|  | WCS6 | 88, 97 | 0.0031 | 852, 743 | 2.67 ± 3.11 | 4.72 ± 7.32 | 0 |
|  | WCS7 | 88, 97 | 0.0035 | 852, 743 | 2.61 ± 2.66 | 0.944 ± 2.24 | 0 |
|  | WCS8 | 88, 97 | 0.0061 | 852, 743 | 2.67 ± 3.31 | 3.61 ± 7.04 | 0 |
| FM 1847 | WCS1 | 88 | 0.0153 | 2753 | 6.45 ± 5.99 | 73 ± 39.5 | 1.7 ± 3.77 |
|  | WCS2 | 88, 105 | 0.0039 | 2050 | 10.7 ± 13.5 | 6.55 ± 10.8 | 0.55 ± 1.88 |
|  | WCS3 | 88, 105 | 0.0005 | 2050 | 6.22 ± 10.4 | 7.33 ± 11 | 0 |
|  | WCS4 | 88, 105 | < 0.0001 | 2050 | 11.8 ± 18.7 | 7.61 ± 18.1 | 0 |
|  | WCS5 | 88, 105 | < 0.0001 | 2050 | 13.6 ± 25.1 | 4.11 ± 5.99 | 0 |
| SH 100 | WCS1 | 105 | 0.0006 | 7048, 7778, 7107 | 6.15 ± 6.56 | 3.25 ± 4.45 | 0 |
|  | WCS2 | 105 | 0.0008 | 7048, 7778, 7107 | 9.25 ± 8.49 | 5 ± 9.31 | 20.4 ± 29 |
|  | WCS3 | 105 | < 0.0001 | 7048, 7778, 7107 | 9.72 ± 5.41 | 0.833 ± 1.54 | 0 |
|  | WCS3A | 105 | 0.0000 | 7189, 7048, 7778, 7107 | 2.97 ± 4.36 | 0.788 ± 3.83 | 0 |
|  | WCS4 | 105 | < 0.0001 | 7048, 7778, 7107 | 5.78 ± 3.9 | 0.222 ± 0.943 | 0 |

^1^Speed limits (km/h) changed from the construction period to the post-construction period. When two numbers are provided, these are the construction and post-construction period speed limits, respectively.

^2^Vehicle traffic information is available from average annual daily traffic (AADT) counts collected by the Texas Department of Transportation yearly. Different values represent year to year changes for the nearest AADT station to the WCS during the monitoring period.

^3^Human, domestic animal, and livestock activity are averaged over the monitoring period. Means ± standard deviation are provided.
