# Supplementary material for: Predicting species assemblages at wildlife crossing structures using multivariate regression of principal coordinates
Source: PLoS One. 2025 Oct 24;20(10):e0335193. doi: 10.1371/journal.pone.0335193 (PMC12551880; doi:10.1371/journal.pone.0335193)
Supplement: S2 Appendix — (DOCX) [file pone.0335193.s002.docx]

**Appendix S2: Description of species detected on camera.**

Table S2.1: Species detected on camera including the common name, scientific name, and species code used in figures throughout the manuscript.

| Common Name | Scientific Name | Species Code |
| --- | --- | --- |
| Nine-banded armadillo | *Dasypus novemcinctus* | ARMA |
| American beaver | *Castor canadensis* | BEAV |
| Bobcat | *Lynx rufus* | BOBC |
| Eastern cottontail | *Sylvilagus floridanus* | ECOT |
| Coyote | *Canis latrans* | COYO |
| Feral hog | *Sus scrofa* | FHOG |
| Fox squirrel | *Sciurus niger* | FSQR |
| Gray squirrel | *Sciurus carolinensis* | GSQR |
| Grey fox | *Urocyon cinereoargenteus* | GFOX |
| Black-tailed jackrabbit | *Lepus californicus* | JACK |
| Javelina | *Pecari tajacu* | JAVE |
| Mexican ground squirrel | *Spermophilus mexicanus* | MSQR |
| Nilgai | *Boselaphus tragocamelus* | NILG |
| Nutria | *Mycastor coypus* | NUTR |
| Ocelot | *Leopardus pardalis* | OCEL |
| Virginia opossum | *Didelphis virginiana* | OPOS |
| Northern raccoon | *Procyon lotor* | RACC |
| Unknown rodent^1^ | *Rodentia spp.* | UROD |
| Striped skunk | *Mephitis mephitis* | SSKU |
| Unknown mammal | *Mammalia spp.* | UMAM |
| Long-tailed weasel | *Mustela frenata* | WEAS |
| White-tailed deer | *Odocoileus virginianus* | DEER |

^1^Rats and mice
