# Supplementary material for: Predicting species assemblages at wildlife crossing structures using multivariate regression of principal coordinates
Source: PLoS One. 2025 Oct 24;20(10):e0335193. doi: 10.1371/journal.pone.0335193 (PMC12551880; doi:10.1371/journal.pone.0335193)
Supplement: S3 Appendix — (DOCX) [file pone.0335193.s003.docx]

**Appendix S3: Detailed results of the model building process showing the model selection process for the spatial, temporal, structural, environmental, and anthropogenic characteristics of wildlife crossing structures (WCSs).**

The minimum spanning tree distance between two WCSs was 6327.9 m while the minimum spanning tree distance between two months equaled one month. We identified eleven possible axes of spatial autocorrelation from dbMEM models. Generally, axes 1-4 represented broad spatial scales while axes 5-11 represented fine spatial scales (Figure S3.1). After model selection, axes 1-10 were included in the model of total detections, axes 1-5 and 7-10 were included in the model of successful crossings, and axes 1-5, 8, and 10 were included in the model of failed crossings (Table S3.1).

There were 22 possible axes of temporal autocorrelation in the dbMEM models. Generally, axes 1-12 represented variability after time interval “-5” which represents the majority of variation among all sites (Figure S3.2). Axes 13-22 represent variability before time interval “-5” which only had data for WCS3A on SH 100. For the total detections model, axes 1, 3, and 13 were included after forward selection. Axes 1, 3-5, and 13 were included in the successful crossings model and axes 1, 2, 5, and 13 were included in the failed crossings model (Table S3.2).

For all models, once spatial and temporal autocorrelation had been accounted for, all structural characteristics and environmental characteristics were retained (Table S3.3, S3.4). For anthropogenic characteristics, domestic activity and livestock activity were excluded from models of total detections and failed crossings. Both predictors were retained in the successful crossings model (Table S3.5).

Table S3.1: Model selection results for the spatial autocorrelation axes using a forward selection procedure based on AICc.

|  | Variable | AICc | SS | Pseudo-F | P | Prop.^1^ | Cumul.^2^ | Res.df^3^ |
| --- | --- | --- | --- | --- | --- | --- | --- | --- |
| Total Detections | PCNM4 | 2562.3 | 41505.0 | 25.93 | < 0.001 | 0.070 | 0.070 | 345 |
|  | +PCNM2 | 2537.9 | 40471.0 | 27.20 | < 0.001 | 0.068 | 0.138 | 344 |
|  | +PCNM1 | 2520.3 | 28186.0 | 19.99 | < 0.001 | 0.047 | 0.186 | 343 |
|  | +PCNM3 | 2503.1 | 26160.0 | 19.56 | < 0.001 | 0.044 | 0.230 | 342 |
|  | +PCNM8 | 2493.7 | 14866.0 | 11.45 | < 0.001 | 0.025 | 0.255 | 341 |
|  | +PCNM5 | 2486.2 | 12049.0 | 9.51 | < 0.001 | 0.020 | 0.275 | 340 |
|  | +PCNM7 | 2479.6 | 10687.0 | 8.63 | < 0.001 | 0.018 | 0.293 | 339 |
|  | +PCNM10 | 2473.8 | 9470.3 | 7.80 | < 0.001 | 0.016 | 0.309 | 338 |
|  | +PCNM9 | 2470.2 | 6687.0 | 5.58 | < 0.001 | 0.011 | 0.320 | 337 |
|  | +PCNM6 | 2469.1 | 3770.6 | 3.17 | 0.003 | 0.006 | 0.326 | 336 |
| Successful Crossings | PCNM2 | 2350.2 | 52271.0 | 24.92 | < 0.001 | 0.076 | 0.076 | 305 |
|  | +PCNM4 | 2334.9 | 35065.0 | 17.63 | < 0.001 | 0.051 | 0.126 | 304 |
|  | +PCNM3 | 2325.2 | 22659.0 | 11.80 | < 0.001 | 0.033 | 0.159 | 303 |
|  | +PCNM10 | 2316.4 | 20236.0 | 10.88 | < 0.001 | 0.029 | 0.188 | 302 |
|  | +PCNM1 | 2310.1 | 15160.0 | 8.35 | < 0.001 | 0.022 | 0.210 | 301 |
|  | +PCNM5 | 2306.0 | 10958.0 | 6.14 | < 0.001 | 0.016 | 0.226 | 300 |
|  | +PCNM7 | 2303.6 | 7808.0 | 4.42 | 0.001 | 0.011 | 0.237 | 299 |
|  | +PCNM8 | 2301.3 | 7581.5 | 4.34 | < 0.001 | 0.011 | 0.248 | 298 |
|  | +PCNM9 | 2300.0 | 5807.3 | 3.35 | 0.005 | 0.008 | 0.257 | 297 |
| Failed Crossings | PCNM4 | 2368.1 | 34010.0 | 15.29 | < 0.001 | 0.048 | 0.048 | 305 |
|  | +PCNM2 | 2354.5 | 33538.0 | 15.82 | < 0.001 | 0.047 | 0.095 | 304 |
|  | +PCNM3 | 2341.7 | 30424.0 | 15.01 | < 0.001 | 0.043 | 0.138 | 303 |
|  | +PCNM5 | 2338.2 | 11021.0 | 5.52 | < 0.001 | 0.015 | 0.153 | 302 |
|  | +PCNM10 | 2335.1 | 10248.0 | 5.20 | < 0.001 | 0.014 | 0.167 | 301 |
|  | +PCNM8 | 2332.0 | 9853.3 | 5.07 | < 0.001 | 0.014 | 0.181 | 300 |
|  | +PCNM1 | 2329.0 | 9713.6 | 5.07 | < 0.001 | 0.014 | 0.195 | 299 |

^1^Proportion of variance explained given that the previous elements were in the model.

^2^Cumulative proportion of variance explained.

^3^Residual degrees of freedom

Table S3.2: Model selection results for the temporal autocorrelation axes using a forward selection procedure based on AICc.

|  | Variable | AICc | SS | Pseudo-F | P | Prop.^1^ | Cumul.^2^ | Res.df^3^ |
| --- | --- | --- | --- | --- | --- | --- | --- | --- |
| Total Detections | PCNM1 | 2579.1 | 14219.0 | 8.46 | < 0.001 | 0.024 | 0.024 | 345 |
|  | +PCNM13 | 2575.3 | 9601.2 | 5.79 | < 0.001 | 0.016 | 0.040 | 344 |
|  | +PCNM3 | 2574.2 | 5108.5 | 3.10 | 0.005 | 0.009 | 0.049 | 343 |
| Successful Crossings | PCNM5 | 2367.9 | 14266.0 | 6.42 | < 0.001 | 0.021 | 0.021 | 305 |
|  | +PCNM4 | 2365.3 | 10164.0 | 4.63 | < 0.001 | 0.015 | 0.035 | 304 |
|  | +PCNM1 | 2363.2 | 8903.7 | 4.10 | 0.001 | 0.013 | 0.048 | 303 |
|  | +PCNM13 | 2361.7 | 7736.9 | 3.59 | 0.004 | 0.011 | 0.059 | 302 |
|  | +PCNM3 | 2360.9 | 6012.6 | 2.81 | 0.012 | 0.009 | 0.068 | 301 |
| Failed Crossings | PCNM5 | 2377.5 | 12853.0 | 5.61 | < 0.001 | 0.018 | 0.018 | 305 |
|  | +PCNM1 | 2374.6 | 11046.0 | 4.88 | < 0.001 | 0.016 | 0.034 | 304 |
|  | +PCNM13 | 2373.4 | 7351.9 | 3.27 | 0.005 | 0.010 | 0.044 | 303 |
|  | +PCNM2 | 2373.4 | 4622.3 | 2.06 | 0.055 | 0.006 | 0.050 | 302 |

^1^Proportion of variance explained given that the previous elements were in the model.

^2^Cumulative proportion of variance explained.

^3^Residual degrees of freedom

Table S3.3: Model selection results for the structural characteristics after spatial and temporal autocorrelation factors had been included.

|  | Variable | AICc | SS | Pseudo-F | P | Prop. | Cumul.^1^ | Res.df^2^ | Reg.df^3^ |
| --- | --- | --- | --- | --- | --- | --- | --- | --- | --- |
| Total Detections | +substrate | 2415.4 | 43655.0 | 21.75 | < 0.001 | 0.074 | 0.441 | 331 | 16 |
|  | +catwalk | 2406.6 | 10373.0 | 10.64 | < 0.001 | 0.017 | 0.458 | 330 | 17 |
|  | +openness | 2405.9 | 2773.7 | 2.86 | 0.006 | 0.005 | 0.463 | 329 | 18 |
|  | +fencing | 2401.9 | 5636.4 | 5.90 | < 0.001 | 0.009 | 0.472 | 328 | 19 |
| Successful Crossings | +substrate | 2262.6 | 45574.0 | 15.25 | < 0.001 | 0.066 | 0.374 | 290 | 17 |
|  | +catwalk | 2256.7 | 11462.0 | 7.85 | < 0.001 | 0.017 | 0.390 | 289 | 18 |
|  | +fencing | 2255.7 | 4460.0 | 3.08 | 0.009 | 0.006 | 0.397 | 288 | 19 |
|  | +openness | 2255.3 | 3557.0 | 2.47 | 0.027 | 0.005 | 0.402 | 287 | 20 |
| Failed Crossings | +substrate | 2293.6 | 55489.0 | 16.59 | < 0.001 | 0.078 | 0.312 | 293 | 14 |
|  | +catwalk | 2293.0 | 4544.8 | 2.73 | 0.013 | 0.006 | 0.318 | 292 | 15 |
|  | +fencing | 2291.9 | 5133.7 | 3.11 | 0.008 | 0.007 | 0.326 | 291 | 16 |
|  | +openness | 2291.7 | 3883.3 | 2.36 | 0.028 | 0.005 | 0.331 | 290 | 17 |

^1^Proportion of variance explained given that the previous elements were in the model.

^2^Residual degrees of freedom.

^3^Regression degrees of freedom

Table S3.4: Model selection results for the environmental characteristics after spatial and temporal autocorrelation factors have been included.

|  | Variable | AICc | SS | Pseudo-F | P | Prop. | Cumul.^1^ | Res.df^2^ | Reg.df^3^ |
| --- | --- | --- | --- | --- | --- | --- | --- | --- | --- |
| Total Detections | +woody | 2431.3 | 25866.0 | 24.54 | < 0.001 | 0.044 | 0.411 | 332 | 15 |
|  | +natural | 2422.0 | 11404.0 | 11.15 | < 0.001 | 0.019 | 0.430 | 331 | 16 |
|  | +water | 2411.2 | 12526.0 | 12.68 | < 0.001 | 0.021 | 0.451 | 330 | 17 |
|  | +precip | 2409.9 | 3228.9 | 3.29 | 0.002 | 0.005 | 0.456 | 329 | 18 |
| Successful Crossings | +woody | 2274.5 | 25140.0 | 16.12 | < 0.001 | 0.036 | 0.344 | 291 | 16 |
|  | +water | 2265.2 | 16849.0 | 11.18 | < 0.001 | 0.024 | 0.369 | 290 | 17 |
|  | +natural | 2259.3 | 11442.0 | 7.77 | < 0.001 | 0.017 | 0.385 | 289 | 18 |
|  | +precip | 2257.8 | 5154.9 | 3.53 | 0.003 | 0.007 | 0.393 | 288 | 19 |
| Failed Crossings | +woody | 2310.3 | 24408.0 | 13.77 | < 0.001 | 0.034 | 0.268 | 294 | 13 |
|  | +water | 2305.7 | 11411.0 | 6.56 | < 0.001 | 0.016 | 0.284 | 293 | 14 |
|  | +natural | 2302.2 | 9456.2 | 5.52 | < 0.001 | 0.013 | 0.298 | 292 | 15 |
|  | +precip | 2300.5 | 6219.1 | 3.66 | 0.002 | 0.009 | 0.307 | 291 | 16 |

^1^Proportion of variance explained given that the previous elements were in the model.

^2^Residual degrees of freedom.

^3^Regression degrees of freedom

Table S3.5: Model selection results for the anthropogenic characteristics after spatial and temporal autocorrelation factors have been included.

|  | Variable | AICc | SS | Pseudo-F | P | Prop. | Cumul.^1^ | Res.df^2^ | Reg.df^3^ |
| --- | --- | --- | --- | --- | --- | --- | --- | --- | --- |
| Total Detections | +traffic | 2428.2 | 29009.0 | 27.77 | < 0.001 | 0.049 | 0.416 | 332 | 15 |
|  | +buildings | 2422.2 | 8085.7 | 7.90 | < 0.001 | 0.014 | 0.430 | 331 | 16 |
|  | +human | 2417.3 | 6852.7 | 6.81 | < 0.001 | 0.012 | 0.441 | 330 | 17 |
|  | +speed | 2413.6 | 5675.3 | 5.72 | < 0.001 | 0.010 | 0.451 | 329 | 18 |
| Successful Crossings | +traffic | 2281.0 | 15397.0 | 9.67 | < 0.001 | 0.022 | 0.330 | 291 | 16 |
|  | +buildings | 2271.3 | 17789.0 | 11.57 | < 0.001 | 0.026 | 0.356 | 290 | 17 |
|  | +speed | 2265.1 | 12013.0 | 8.00 | < 0.001 | 0.017 | 0.373 | 289 | 18 |
|  | +domestic | 2264.3 | 4325.2 | 2.90 | 0.010 | 0.006 | 0.380 | 288 | 19 |
|  | +human | 2263.7 | 4095.1 | 2.76 | 0.013 | 0.006 | 0.385 | 287 | 20 |
|  | +livestock | 2263.5 | 3506.1 | 2.38 | 0.032 | 0.005 | 0.391 | 286 | 21 |
| Failed Crossings | +traffic | 2312.2 | 21247.0 | 11.92 | < 0.001 | 0.030 | 0.264 | 294 | 13 |
|  | +buildings | 2303.7 | 17923.0 | 10.37 | < 0.001 | 0.025 | 0.289 | 293 | 14 |
|  | +human | 2298.9 | 11460.0 | 6.76 | < 0.001 | 0.016 | 0.305 | 292 | 15 |
|  | +speed | 2298.5 | 4168.4 | 2.47 | 0.021 | 0.006 | 0.311 | 291 | 16 |

^1^Proportion of variance explained given that the previous elements were in the model.

^2^Residual degrees of freedom.

^3^Regression degrees of freedom


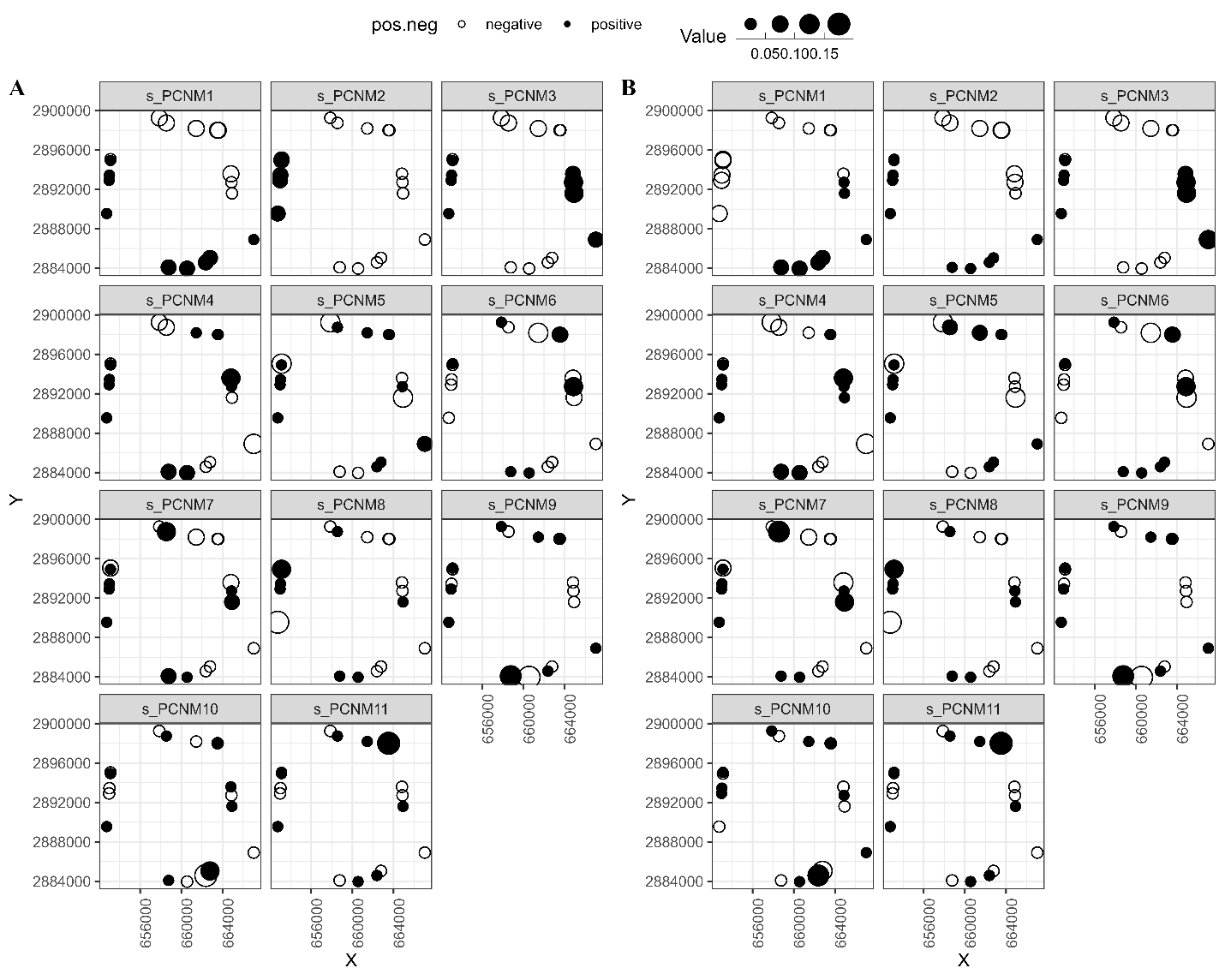


Figure S3.1: Figures of the dbMEM axes vs the coordinates to identify the scale of effect for spatial autocorrelation for total detections (A) and successful/unsuccessful interactions (B).


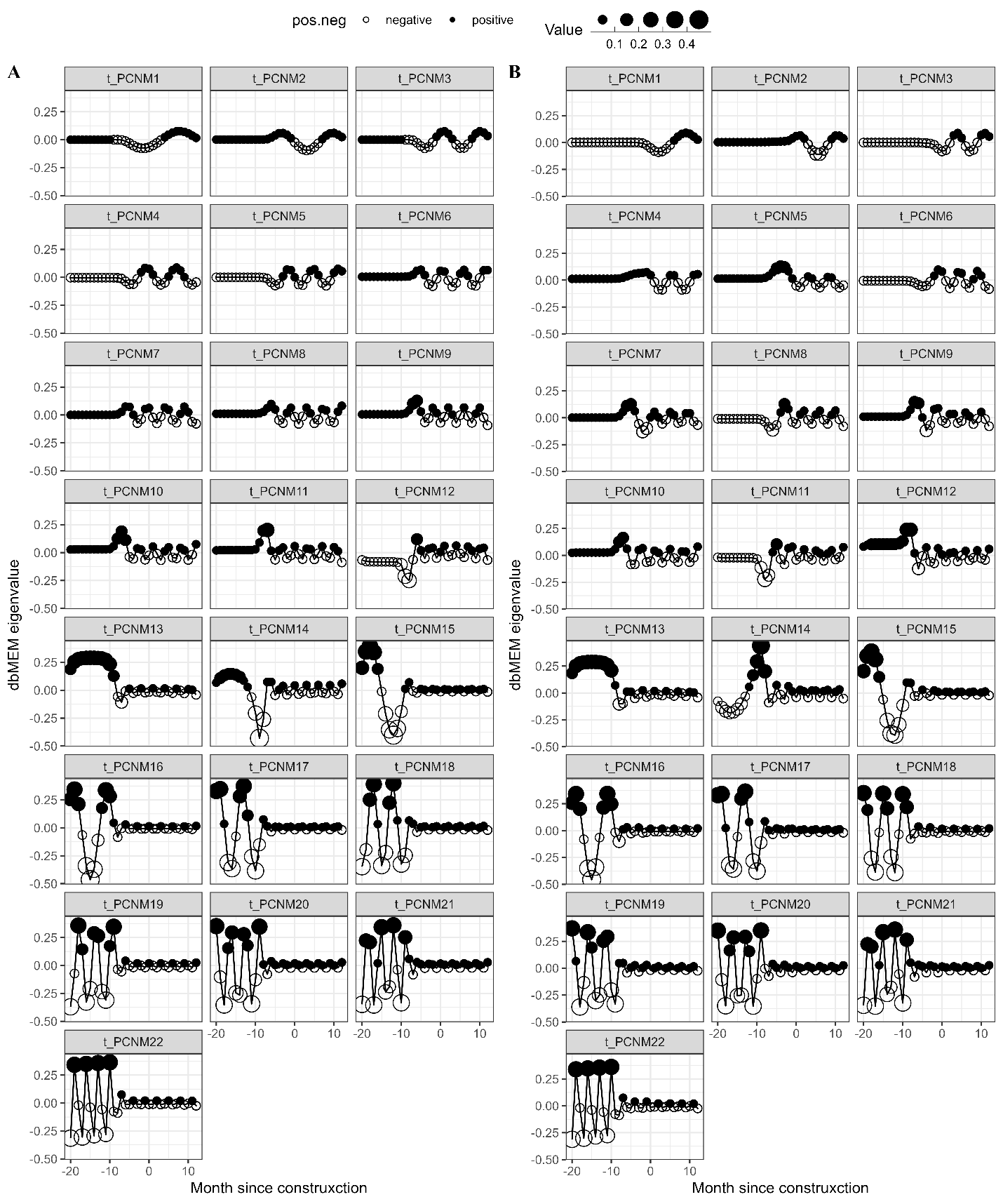


Figure S3.2: Figures of the dbMEM axes vs month to identify the scale of effect for temporal autocorrelation for total detections (A) and successful/unsuccessful interactions (B).
