# Supplementary material for: Predicting species assemblages at wildlife crossing structures using multivariate regression of principal coordinates
Source: PLoS One. 2025 Oct 24;20(10):e0335193. doi: 10.1371/journal.pone.0335193 (PMC12551880; doi:10.1371/journal.pone.0335193)
Supplement: S4 Appendix — (DOCX) [file pone.0335193.s004.docx]

**Appendix S4: Variation partitioning model construction and results**

Below we provide the code, created in R v4.3.1 to conduct variation partitioning with five sets of variables. This is an expansion on the *varpart* function in the *vegan* package in R (Oksanen et al. 2018). Note that unlike the *varpart* function which calculates them internally, we calculated adjusted R^2^ values using the Program Primer v7 (Primer-E, Albany, Auckland, New Zealand) to ensure that the appropriate resemblance matrix was used with our data. The function imports the R^2^ values from Primer and calculates the variation partitioning results.

varpart5 <- function(parts, Xs, type){

#parts <- vp2$total.detections

#Xs <- c("str", "env", "ant", "spat", "temp")

#type <- "AdjustedR2"

# Define the sets

names(Xs) <- paste("X", 1:length(Xs), sep = "")

Xs2 <- data.frame(t(Xs))

# The original output RDA values

ps1 <- as.list(parts[,type])

names(ps1) <- parts$RDA

# Intermediary functions

Is <- with(ps1, list(A29 - A25,

A28 - A24,

A27 - A23,

A26 - A22,

A28 - A21,

A27 - A20,

A26 - A19,

A27 - A18,

A27 - A17,

A26 - A16,

A21 - A15,

A20 - A14,

A19 - A13,

A18 - A12,

A17 - A11,

A16 - A10,

A18 - A9,

A17 - A8,

A16 - A7,

A16 - A6,

A9 - A5,

A8 - A4,

A7 - A3,

A6 - A2,

A6 - A1))

names(Is) <- paste("I", seq(1,length(Is)), sep = "")

# Computing the proportion of variance explained

## Single set and two set partitions

ps2 <- with(ps1, with(Is, list(A31 - A30,

A31 - A29,

A31 - A28,

A31 - A27,

A31 - A26,

I1 - A1,

I2 - A1,

I3 - A1,

I4 - A1,

I5 - A2,

I6 - A2,

I7 - A2,

I8 - A3,

I9 - A3,

I10 - A4)))

names(ps2) <- paste("B", seq(1,length(ps2)), sep = "")

## 3 set partitions

ps2b <- with(ps1, with(Is, with(ps2, list(I11 - I1 - B7,

I12 - I1 - I8,

I13 - I1 - B9,

I14 - I2 - B8,

I15 - I2 - B9,

I16 - I3 - B9,

I17 - I5 - B11,

I18 - I5 - B12,

I19 - I6 - B12,

I20 - I8 - B14))))

names(ps2b) <- paste("B", seq(length(ps2)+1,length(ps2) + length(ps2b)), sep = "")

ps2 <- c(ps2, ps2b)

## 4 set partitions

ps2c <- with(ps1, with(Is, with(ps2, list(I21 - I11 - B8 - B17 - B19,

I22 - I11 - B9 - B18 - B20,

I23 - I12 - B9 - B20 - B21,

I24 - I17 - B12 - B23 - B24,

I25 - I17 - B12 - B23 - B24))))

names(ps2c) <- paste("B", seq(length(ps2) + 1, length(ps2) + length(ps2c)), sep = "")

ps2 <- c(ps2, ps2c)

## 5 set partitions

ps2d <- with(ps1, with(Is, with(ps2, list(A1 - I11 - B8 - B9 - B17 - B18 - B19 - B20 - B21 - B26 - B27 - B28 - B29,

1 - A31))))

names(ps2d) <- paste("B", seq(length(ps2) + 1, length(ps2) + length(ps2d)), sep = "")

ps2 <- c(ps2, ps2d)

# The groups that each set belongs to

Bs <- with(Xs2, list(X1, X2, X3, X4, X5,

c(X1, X2), c(X1, X3), c(X1, X4), c(X1, X5),

c(X2, X3), c(X2, X4), c(X2, X5),

c(X3, X4), c(X3, X5),

c(X4, X5),

c(X1, X2, X3), c(X1, X2, X4), c(X1, X2, X5), c(X1, X3, X4), c(X1, X3, X5), c(X1, X4, X5),

c(X2, X3, X4), c(X2, X3, X5), c(X2, X4, X5), c(X3, X4, X5),

c(X1, X2, X3, X4), c(X1, X2, X3, X5), c(X1, X2, X4, X5), c(X1, X3, X4, X5), c(X2, X3, X4, X5),

c(X1, X2, X3, X4, X5),

"residual"

)

)

# Output as a data.frame

out <- data.frame(name = names(ps2),

no.sets = c(sapply(Bs[-length(Bs)], length), 0),

partition = sapply(Bs, function(x){paste(x, collapse = " ")}),

type = do.call(c, ps2))

colnames(out) <- c("name", "no.sets", "partition", type)

row.names(out) <- NULL

return(out)

}

Table S4.1: Proportion of the explained variation of each for five sets of predictors (Str = structural, Env = environmental, Ant = anthropogenic, Spat = spatial, Temp = temporal) and their interactions for total detections.

| Set | Adj. R^2^ | Set | Adj. R^2^ | Set | Adj. R^2^ |
| --- | --- | --- | --- | --- | --- |
| **One set** |  | **Three sets** |  | **Four sets** |  |
| Spat | 0.096 | Spat + |  | Spat + |  |
| Temp | 0.016 | Temp + |  | Temp + |  |
| Str | 0.013 | Str | 0.163 | Str + |  |
| Env | 0.014 | Env | 0.086 | Env | -0.158 |
| Ant | 0.025 | Ant | 0.175 | Ant | -0.015 |
|  |  | Str + |  | Env + |  |
| **Two sets** |  | Env | 0.055 | Ant | -0.084 |
| Spat + |  | Ant | 0.174 | Str + |  |
| Temp | -0.208 | Env + |  | Env + |  |
| Str | -0.040 | Ant | 0.118 | Ant | -0.117 |
| Env | -0.042 | Temp + |  | Temp + |  |
| Ant | -0.038 | Str + |  | Str + |  |
| Temp + |  | Env | 0.155 | Env + |  |
| Str | -0.158 | Ant | 0.158 | Ant | -0.149 |
| Env | -0.085 | Env + |  |  |  |
| Ant | -0.161 | Ant | 0.086 | **Five sets** |  |
| Str + |  | Str + |  | Spat + |  |
| Env | -0.125 | Env + |  | Temp + |  |
| Ant | -0.142 | Ant | 0.188 | Str + |  |
| Env + |  |  |  | Env + |  |
| Ant | -0.080 |  |  | Ant | 0.009 |
|  |  |  |  | Residual | 0.518 |

Table S4.2: Proportion of the explained variation for each of five sets of predictors (Str = structural, Env = environmental, Ant = anthropogenic, Spat = spatial, Temp = temporal) and their interactions for successful crossings.

| Set | Adj. R^2^ | Set | Adj. R^2^ | Set | Adj. R^2^ |
| --- | --- | --- | --- | --- | --- |
| **One set** |  | **Three sets** |  | **Four sets** |  |
| Spat | 0.096 | Spat + |  | Spat + |  |
| Temp | 0.042 | Temp + |  | Temp + |  |
| Str | 0.043 | Str | 0.100 | Str + |  |
| Env | 0.039 | Env | 0.040 | Env | -0.096 |
| Ant | 0.032 | Ant | 0.116 | Ant | 0.020 |
|  |  | Str + |  | Env + |  |
| **Two sets** |  | Env | 0.010 | Ant | -0.037 |
| Spat + |  | Ant | 0.088 | Str + |  |
| Temp | -0.143 | Env + |  | Env + |  |
| Str | -0.023 | Ant | 0.038 | Ant | -0.033 |
| Env | -0.016 | Temp + |  | Temp + |  |
| Ant | 0.006 | Str + |  | Str + |  |
| Temp + |  | Env | 0.093 | Env + |  |
| Str | -0.098 | Ant | 0.097 | Ant | -0.090 |
| Env | -0.044 | Env + |  |  |  |
| Ant | -0.112 | Ant | 0.042 | **Five sets** |  |
| Str + |  | Str + |  | Spat + |  |
| Env | -0.079 | Env + |  | Temp + |  |
| Ant | -0.078 | Ant | 0.105 | Str + |  |
| Env + |  |  |  | Env + |  |
| Ant | -0.025 |  |  | Ant | -0.016 |
|  |  |  |  | Residual | 0.556 |

Table S4.3: Proportion of the explained variation for each of five sets of predictors (Str = structural, Env = environmental, Ant = anthropogenic, Spat = spatial, Temp = temporal) and their interactions for failed crossings.

| Set | Adj. R^2^ | Set | Adj. R^2^ | Set | Adj. R^2^ |
| --- | --- | --- | --- | --- | --- |
| **One set** |  | **Three sets** |  | **Four sets** |  |
| Spat | 0.062 | Spat + |  | Spat + |  |
| Temp | 0.016 | Temp + |  | Temp + |  |
| Str | 0.035 | Str | 0.117 | Str + |  |
| Env | 0.024 | Env | 0.039 | Env | -0.115 |
| Ant | 0.040 | Ant | 0.059 | Ant | 0.050 |
|  |  | Str + |  | Env + |  |
| **Two sets** |  | Env | 0.061 | Ant |  |
| Spat + |  | Ant | 0.113 | Str + | -0.037 |
| Temp | -0.112 | Env + |  | Env + |  |
| Str | -0.016 | Ant | 0.062 | Ant |  |
| Env | -0.029 | Temp + |  | Temp + | -0.098 |
| Ant | 0.005 | Str + |  | Str + |  |
| Temp + |  | Env | 0.113 | Env + |  |
| Str | -0.116 | Ant | 0.116 | Ant | -0.109 |
| Env | -0.039 | Env + |  |  |  |
| Ant | -0.061 | Ant | 0.038 | **Five sets** |  |
| Str + |  | Str + |  | Spat + |  |
| Env | -0.095 | Env + |  | Temp + |  |
| Ant | -0.107 | Ant | 0.140 | Str + |  |
| Env + |  |  |  | Env + |  |
| Ant | -0.041 |  |  | Ant | -0.054 |
|  |  |  |  | Residual | 0.666 |
