# Supplementary material for: Predicting species assemblages at wildlife crossing structures using multivariate regression of principal coordinates
Source: PLoS One. 2025 Oct 24;20(10):e0335193. doi: 10.1371/journal.pone.0335193 (PMC12551880; doi:10.1371/journal.pone.0335193)
Supplement: S5 Appendix — (DOCX) [file pone.0335193.s005.docx]

**Appendix S5: Model selection and validation results for the full and predictive models**

By detrending the autocorrelation, we found adjusted R^2^ values of 51.82% compared to 46.74% by not detrending the autocorrelation in the total detections analysis. Our detrended model had an adjusted R^2^ value of 49.46% in the successful crossings model, while the non-detrended model was 40.66%. For failed crossings, the adjusted R^2^ was 37.97% by detrending autocorrelation and 34.41% when not detrending autocorrelation. The Mantel correlations of the non-detrended models were 3-9% lower than the detrended models.

We identified up to 20, 13, and 15 non-trivial PCO axes for total detections (Figure S5.1), successful crossings (Figure S5.2), and failed crossings (Figure S5.3), respectively, using the five methods although methods 1, 3, 4, and 5 showed only three non-trivial axes for total detections and successful crossings and two non-trivial axes for failed crossings so we examined the first three axes for total detections and successful crossings and two axes for failed crossings (Figure S5.1, S5.2, S5.3). The full, non-detrended model had R^2^ values of 0.65, 0.67, and 0.40 respectively for the first three PCO axes of total detections, 0.38, 0.54, and 0.38 for the first three PCO axes of successful crossings, and 0.45 and 0.33 for the first two PCO axes of failed crossings. We sufficiently satisfied the assumptions of the permutation tests although some lack of independence was observed, likely due to not detrending the spatial and temporal autocorrelation (Figure S5.4, S5.5, S5.6).

When we iteratively dropped one WCS and predicted that site using the fitted model, we achieved average Mantel correlations between the observed PCO axes and the predicted PCO scores (calculation 3) of 0.30 ± 0.24 (range -0.07 to 0.60) for total detections, 0.21 ± 0.17 (range -0.08 to 0.53) for successful crossings, and 0.17 ± 0.25 (range -0.14 to 0.65) for failed crossings (Tables S5.1, S5.2, and S5.3). Upon visual inspection of the predicted ordination diagrams (Figures 3, 4, 5, Appendix S6), total detections were generally well predicted while successful and failed crossings had mixed results. WCS4 on SH 100 was the only site to be poorly predicted by the total detections model (Figure 3). WCS1 and WCS2 on FM 1847, WCS2 and WCS4 on SH 100, and WCS7 and WCS8 on FM 106 were poorly predicted by the successful crossings model (Figure 4). The failed crossings model poorly predicted WCS1 and WCS2 on FM 1847, WCS2 on SH 100, and WCS8 on FM 106 (Figure 5).

Table S5.1: Mantel correlations assessing the model fit of the predictive model for all drop-one-site models for the total detections model showing the correlation between the original data and the fitted data from the full model (M1; this does not change across models), the correlation between the full drop.one model and the fitted data from that model (M2), the correlation between the original data from the full model and predicted value from the drop-one-site model (M3), the correlation between the fitted data from the full model and predicted value from the drop-one-site model (M4), and the correlation between the full original data and the fitted data from the drop-one-site model (M5).

| WCS | M1 | M2 | M3 | M4 | M5 |
| --- | --- | --- | --- | --- | --- |
| FM106_WCS1 | 0.622 | 0.623 | 0.133 | 0.996 | 0.126 |
| FM106_WCS2 | 0.622 | 0.628 | 0.580 | 0.982 | 0.142 |
| FM106_WCS3 | 0.622 | 0.647 | 0.601 | 0.985 | 0.146 |
| FM106_WCS4 | 0.622 | 0.630 | 0.510 | 0.995 | 0.148 |
| FM106_WCS5 | 0.622 | 0.624 | 0.214 | 0.999 | 0.142 |
| FM106_WCS6 | 0.622 | 0.636 | 0.356 | 0.994 | 0.165 |
| FM106_WCS7 | 0.622 | 0.632 | 0.536 | 0.979 | 0.165 |
| FM106_WCS8 | 0.622 | 0.627 | 0.470 | 0.995 | 0.201 |
| FM1847_WCS1 | 0.622 | 0.607 | 0.604 | 0.988 | 0.216 |
| FM1847_WCS2 | 0.622 | 0.633 | 0.124 | 0.974 | 0.287 |
| FM1847_WCS3 | 0.622 | 0.621 | 0.141 | 0.997 | 0.329 |
| FM1847_WCS4 | 0.622 | 0.639 | -0.018 | 0.992 | 0.334 |
| FM1847_WCS5 | 0.622 | 0.620 | 0.047 | 0.998 | 0.347 |
| SH100_WCS1 | 0.622 | 0.590 | -0.066 | 0.996 | 0.380 |
| SH100_WCS2 | 0.622 | 0.647 | 0.396 | 0.994 | 0.430 |
| SH100_WCS3 | 0.622 | 0.620 | 0.057 | 0.993 | 0.532 |
| SH100_WCS3A | 0.622 | 0.618 | 0.103 | 0.982 | 0.572 |
| SH100_WCS4 | 0.622 | 0.630 | 0.590 | 0.959 | 0.566 |

Table S5.2: Mantel correlations assessing the model fit of the predictive model for all drop-one-site models for the successful crossings model showing the correlation between the full original data and the fitted data from the full model (M1; this does not change across models), the correlation between the full drop-one-site model and the fitted data from that model (M2), the correlation between the original data from the full model and predicted value from the drop-one-site model (M3), the correlation between the fitted data from the full model and predicted value from the drop-one-site model (M4), and the correlation between the full original data and the fitted data from the drop-one-site model (M5).

| WCS | M1 | M2 | M3 | M4 | M5 |
| --- | --- | --- | --- | --- | --- |
| FM106_WCS1 | 0.462 | 0.450 | 0.126 | 0.998 | 0.096 |
| FM106_WCS2 | 0.462 | 0.448 | 0.054 | 0.996 | 0.096 |
| FM106_WCS3 | 0.462 | 0.476 | 0.125 | 0.998 | 0.085 |
| FM106_WCS4 | 0.462 | 0.462 | 0.445 | 0.993 | 0.126 |
| FM106_WCS5 | 0.462 | 0.459 | 0.319 | 0.992 | 0.134 |
| FM106_WCS6 | 0.462 | 0.460 | 0.127 | 0.990 | 0.145 |
| FM106_WCS7 | 0.462 | 0.466 | 0.163 | 0.997 | 0.152 |
| FM106_WCS8 | 0.462 | 0.452 | 0.095 | 0.924 | 0.159 |
| FM1847_WCS1 | 0.462 | 0.481 | 0.205 | 0.903 | 0.118 |
| FM1847_WCS2 | 0.462 | 0.514 | 0.049 | 0.974 | 0.114 |
| FM1847_WCS3 | 0.462 | 0.476 | 0.205 | 0.980 | 0.271 |
| FM1847_WCS4 | 0.462 | 0.485 | 0.195 | 0.985 | 0.267 |
| FM1847_WCS5 | 0.462 | 0.483 | 0.532 | 0.997 | 0.298 |
| SH100_WCS1 | 0.462 | 0.442 | -0.075 | 0.997 | 0.301 |
| SH100_WCS2 | 0.462 | 0.455 | 0.487 | 0.869 | 0.291 |
| SH100_WCS3 | 0.462 | 0.516 | 0.040 | 0.957 | 0.395 |
| SH100_WCS3A | 0.462 | 0.484 | 0.362 | 0.953 | 0.323 |
| SH100_WCS4 | 0.462 | 0.490 | 0.360 | 0.902 | 0.326 |

Table S5.3: Mantel correlations assessing the model fit of the predictive model for all drop-one-site models for the failed crossings model showing the correlation between the full original data and the fitted data from the full model (M1; this does not change across models), the correlation between the full drop-one-site model and the fitted data from that model (M2), the correlation between the original data from the full model and predicted value from the drop-one-site model (M3), the correlation between the fitted data from the full model and predicted value from the drop-one-site model (M4), and the correlation between the full original data and the fitted data from the drop-one-site model (M5).

| WCS | M1 | M2 | M3 | M4 | M5 |
| --- | --- | --- | --- | --- | --- |
| FM106_WCS1 | 0.439 | 0.454 | 0.080 | 0.884 | 0.118 |
| FM106_WCS2 | 0.439 | 0.424 | 0.133 | 0.990 | 0.112 |
| FM106_WCS3 | 0.439 | 0.452 | 0.291 | 0.997 | 0.101 |
| FM106_WCS4 | 0.439 | 0.457 | 0.583 | 0.987 | 0.140 |
| FM106_WCS5 | 0.439 | 0.451 | 0.645 | 0.988 | 0.130 |
| FM106_WCS6 | 0.439 | 0.439 | 0.296 | 0.999 | 0.139 |
| FM106_WCS7 | 0.439 | 0.439 | 0.254 | 0.998 | 0.139 |
| FM106_WCS8 | 0.439 | 0.428 | 0.099 | 0.955 | 0.131 |
| FM1847_WCS1 | 0.439 | 0.466 | 0.077 | 0.984 | 0.081 |
| FM1847_WCS2 | 0.439 | 0.449 | -0.070 | 0.890 | 0.160 |
| FM1847_WCS3 | 0.439 | 0.433 | -0.021 | 0.893 | 0.182 |
| FM1847_WCS4 | 0.439 | 0.472 | 0.162 | 0.708 | 0.171 |
| FM1847_WCS5 | 0.439 | 0.462 | -0.135 | 0.992 | 0.202 |
| SH100_WCS1 | 0.439 | 0.379 | -0.136 | 0.994 | 0.236 |
| SH100_WCS2 | 0.439 | 0.440 | 0.258 | 0.988 | 0.277 |
| SH100_WCS3 | 0.439 | 0.440 | -0.142 | 0.969 | 0.345 |
| SH100_WCS3A | 0.439 | 0.431 | 0.032 | 0.974 | 0.342 |
| SH100_WCS4 | 0.439 | 0.460 | 0.607 | 0.946 | 0.402 |


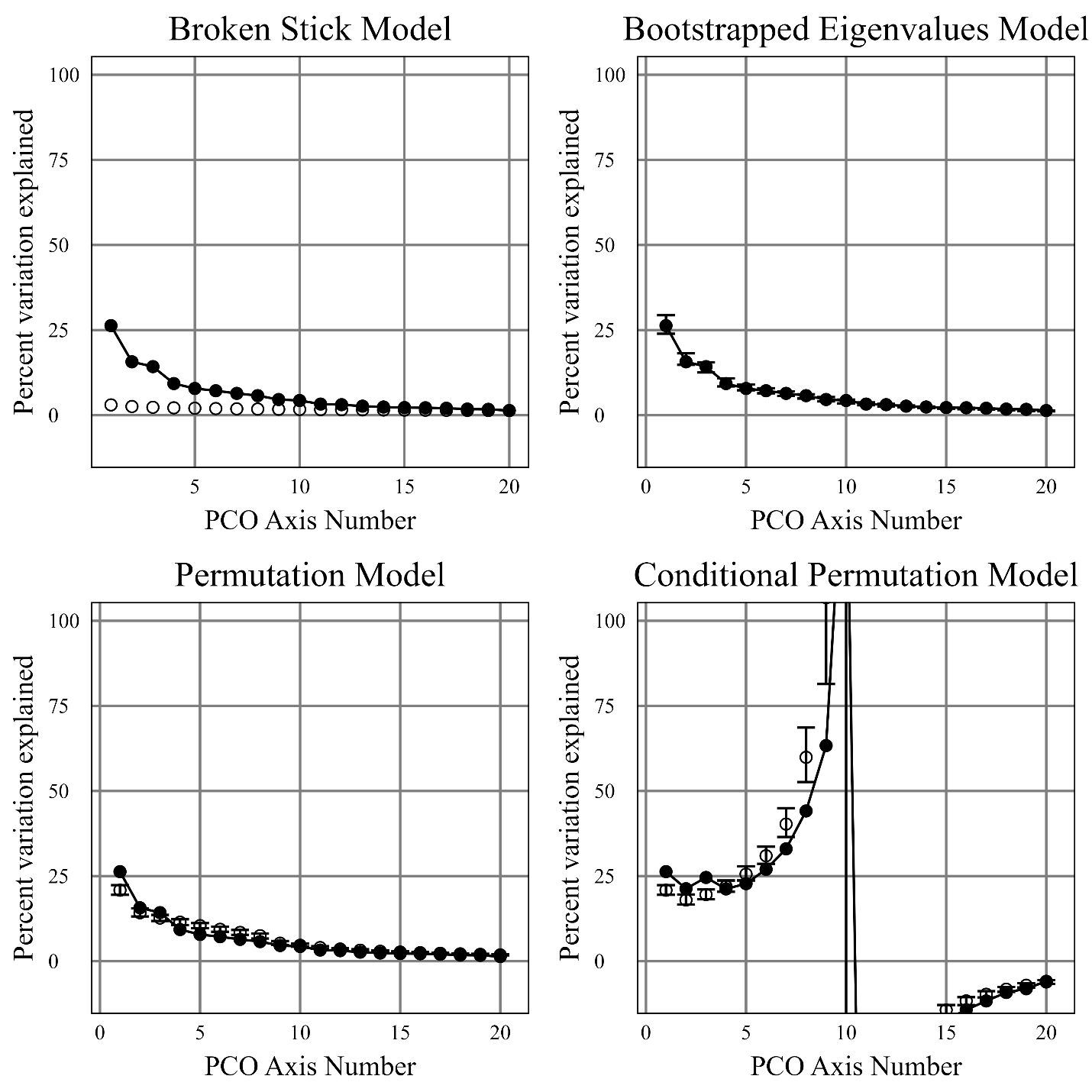


Figure S5.1: Scree plots to identify non-trivial principal coordinates axes for the total detections model.


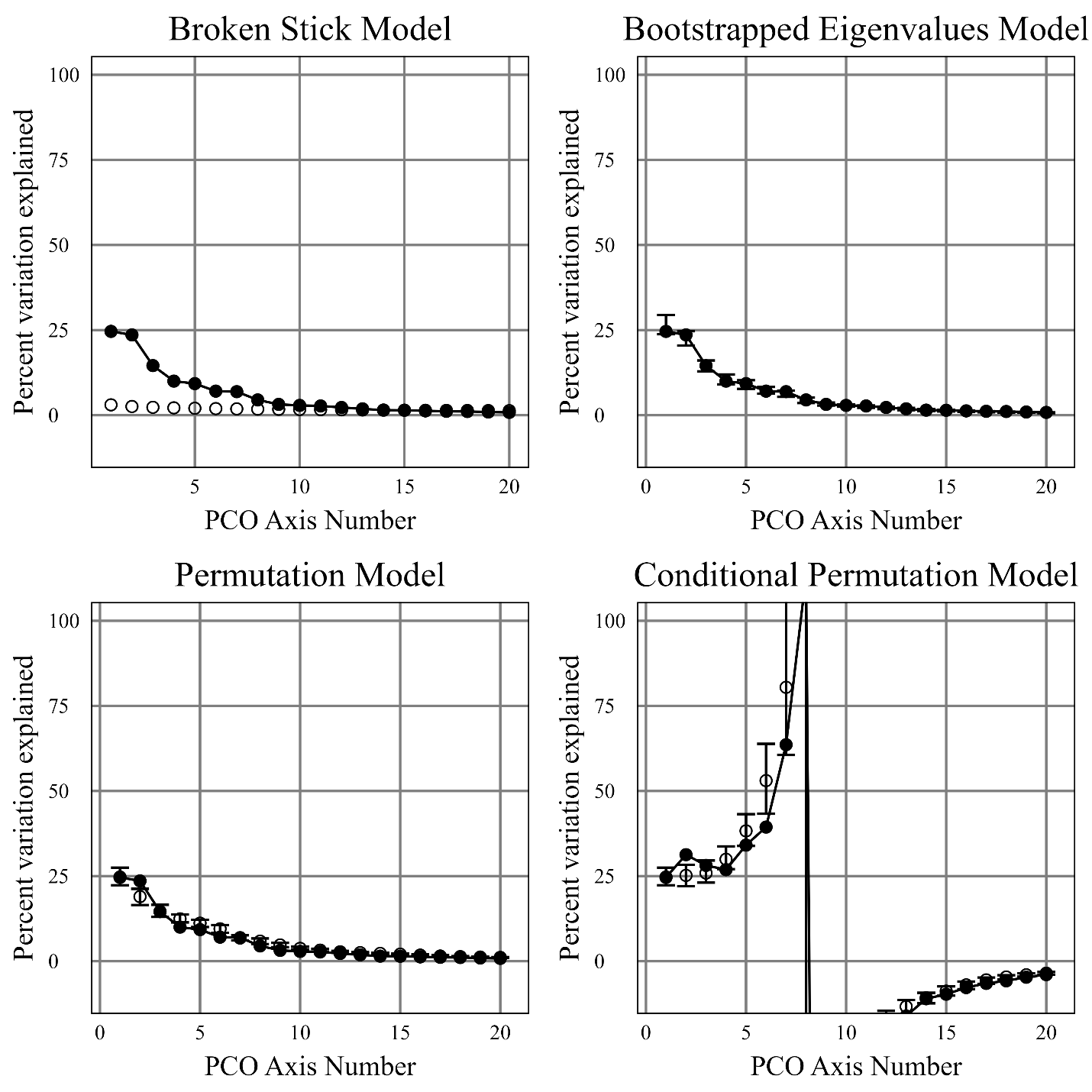


Figure S5.2: Scree plots to identify non-trivial principal coordinates axes for the successful crossings model.


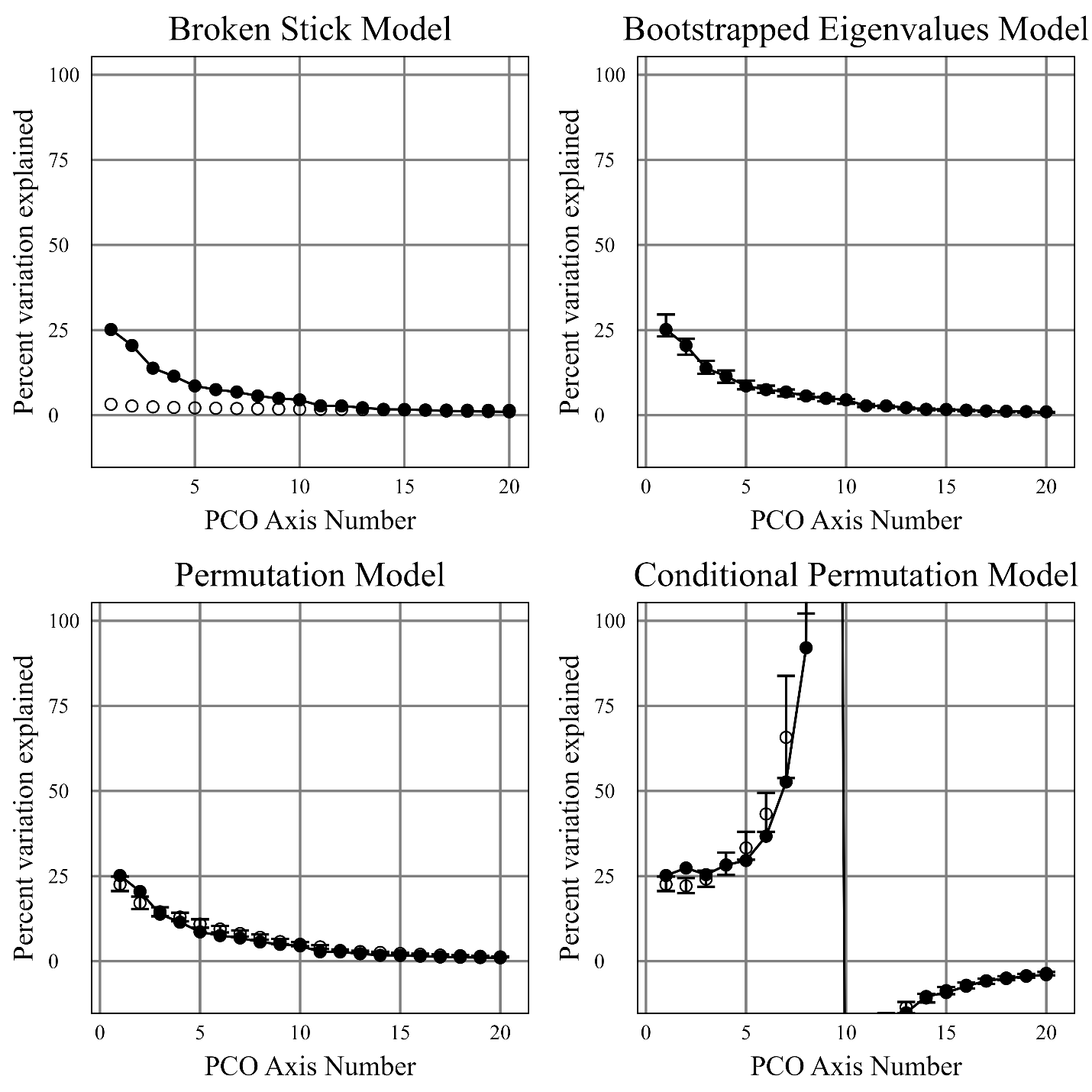


Figure S5.3: Scree plots to identify non-trivial principal coordinates axes for the failed crossings model.


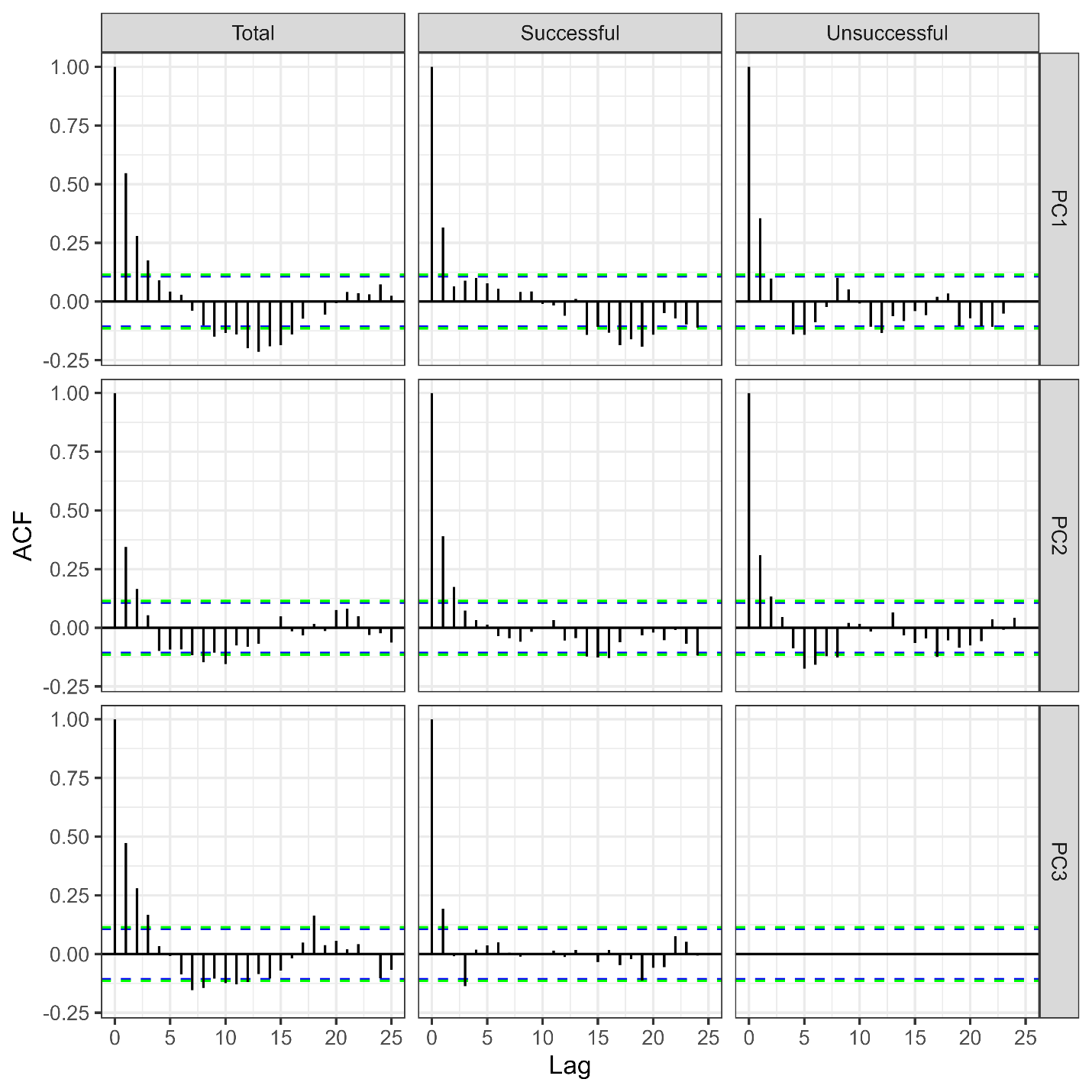


Figure S5.4. Autocorrelation functions (ACF) for total detections, successful crossings, and failed crossings along principal coordinates (PC) axes 1, 2, and 3 used to assess the assumption of independence of errors for the permutation tests of distance-based redundancy analysis.


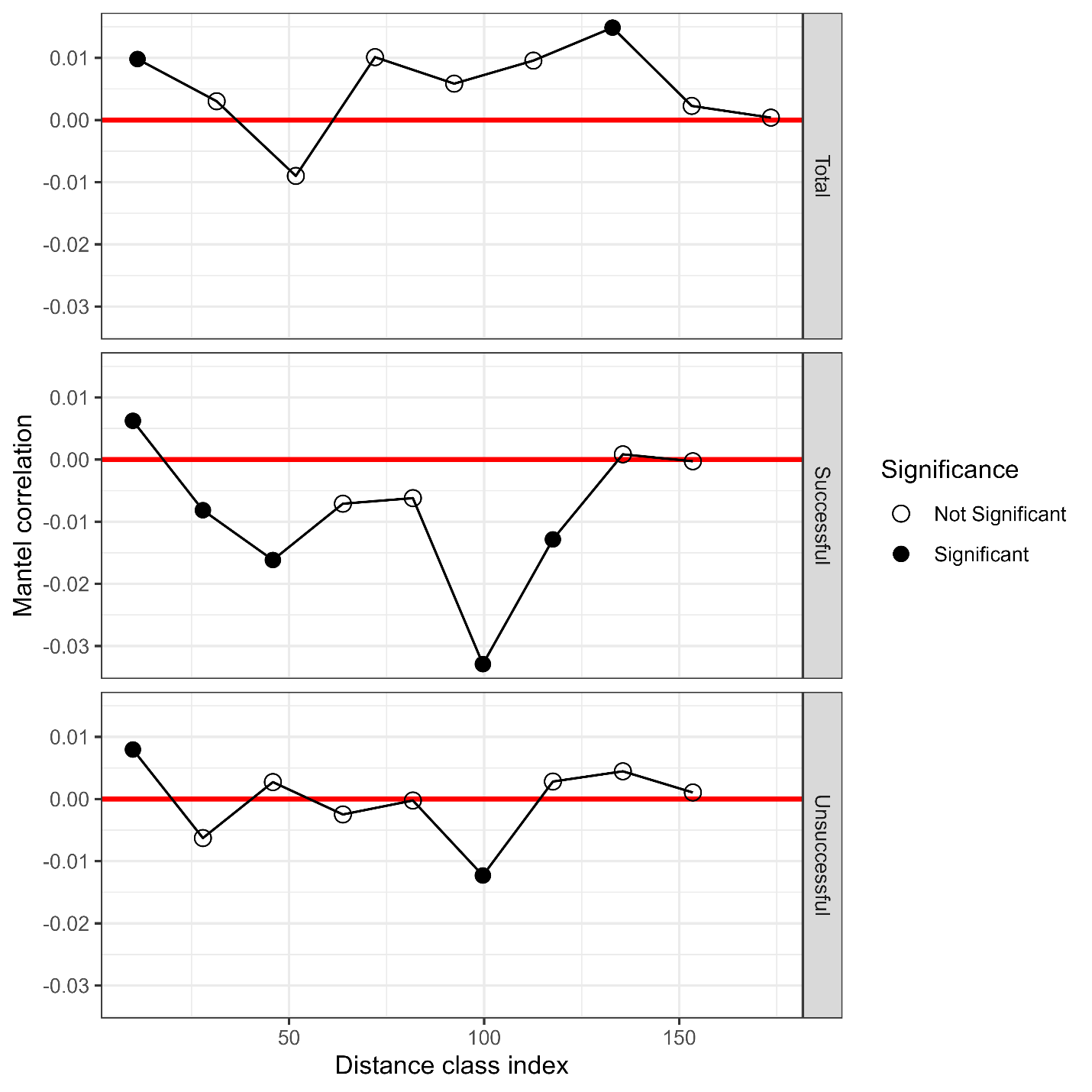


Figure S5.5: Mantel correlograms for total detections, successful crossings, and failed crossings along principal coordinates (PC) axes 1, 2, and 3 used to assess the assumption of independence of errors for the permutation tests of distance-based redundancy analysis.


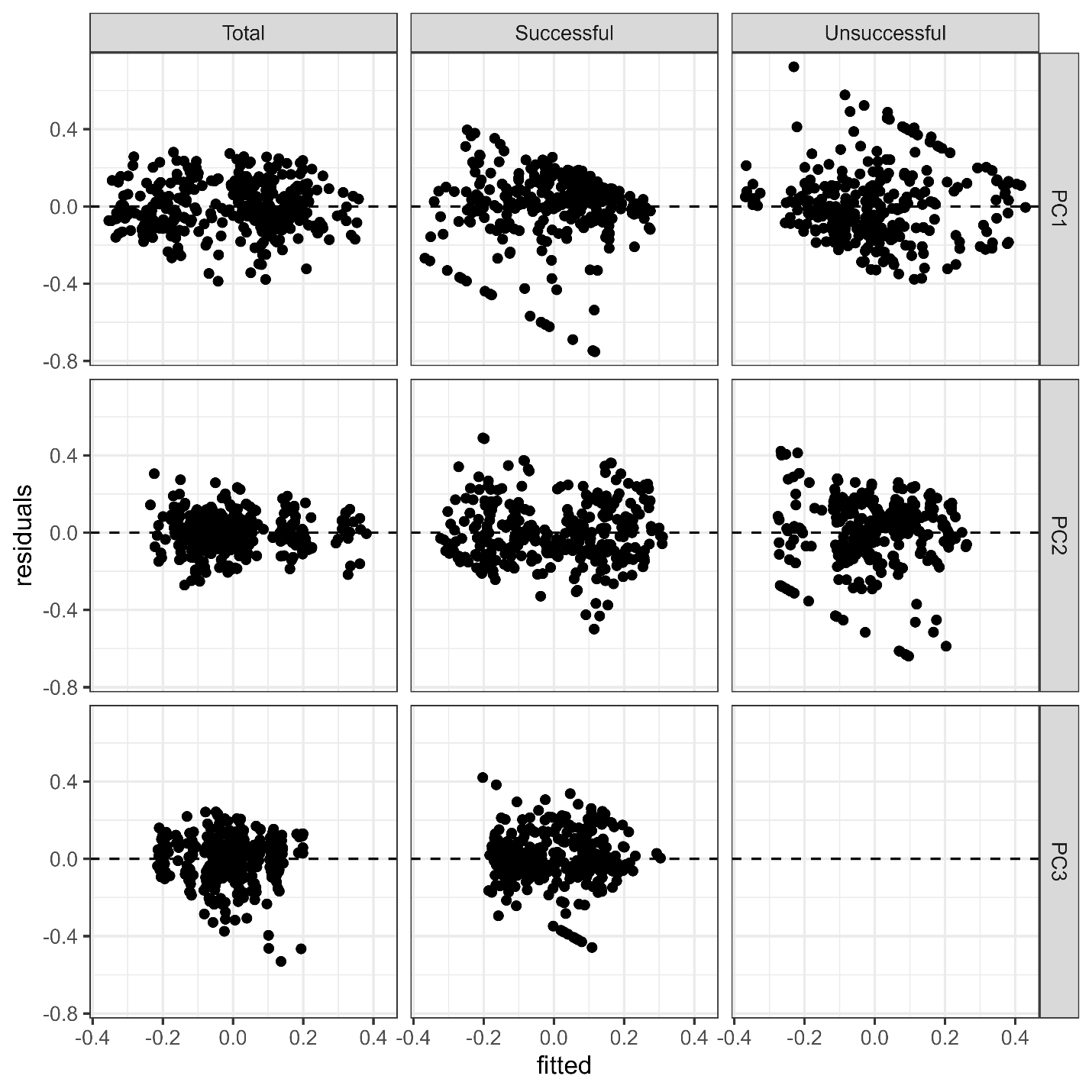


Figure S5.6: Fitted principal coordinate (PC) axes vs residuals for total detections, successful crossings, and failed crossings along PC axes 1, 2, and 3 used to assess the assumption of identically distributed errors for the permutation tests of distance-based redundancy analysis.


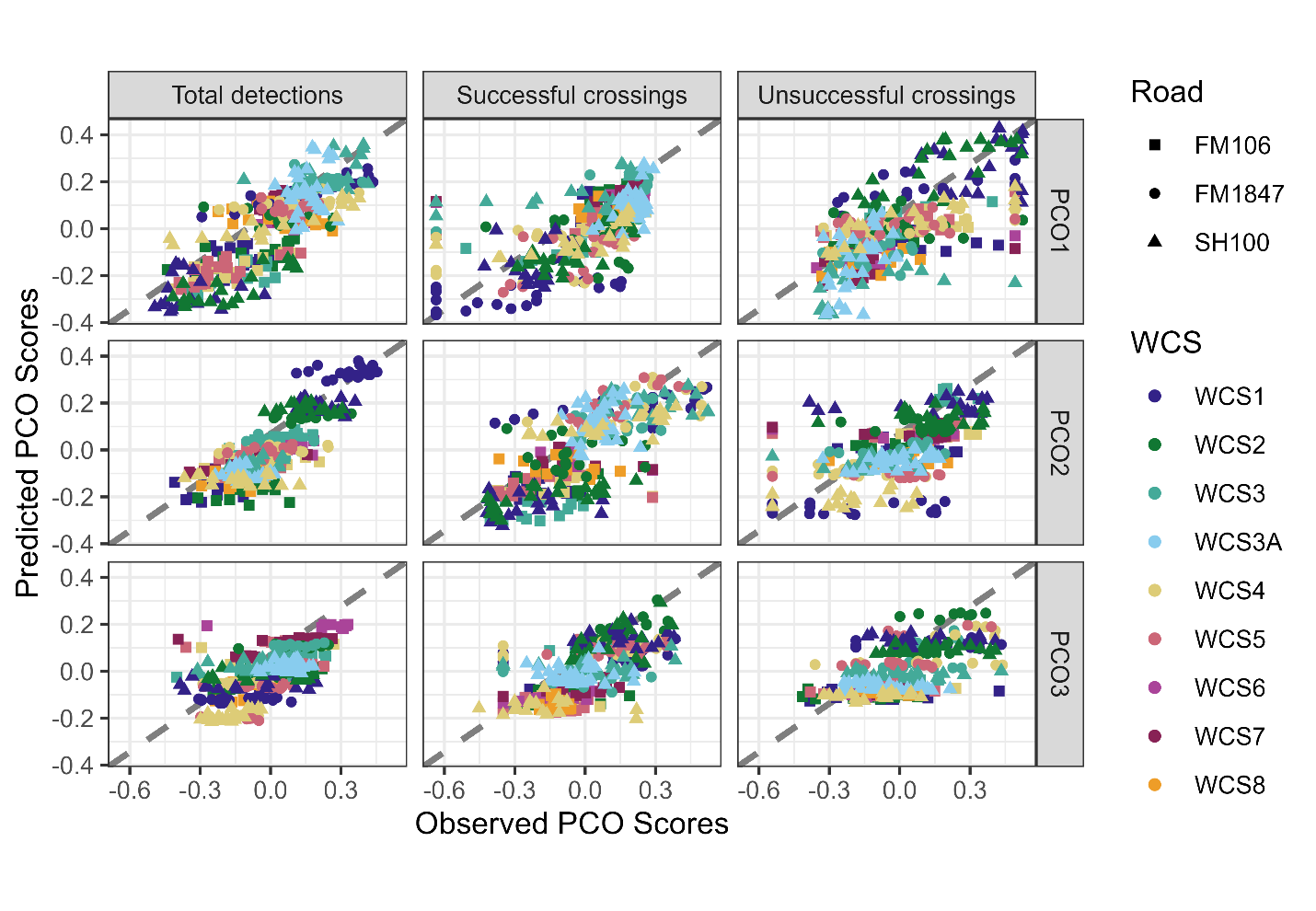


Figure S5.7: Figures of the predicted axes vs the observed axes for all sites and relevant PCO axes from the drop-one-site models.
