# Supplementary material for: Predicting species assemblages at wildlife crossing structures using multivariate regression of principal coordinates
Source: PLoS One. 2025 Oct 24;20(10):e0335193. doi: 10.1371/journal.pone.0335193 (PMC12551880; doi:10.1371/journal.pone.0335193)
Supplement: S6 Appendix — (DOCX) [file pone.0335193.s006.docx]

**Appendix S6: Ordination diagrams from the distance-based redundancy analysis results.**

Figures S6.1-S6.3: Bi-plots of axes 1 and 2 showing the correlations between the site scores and the species scores and predictor variables.
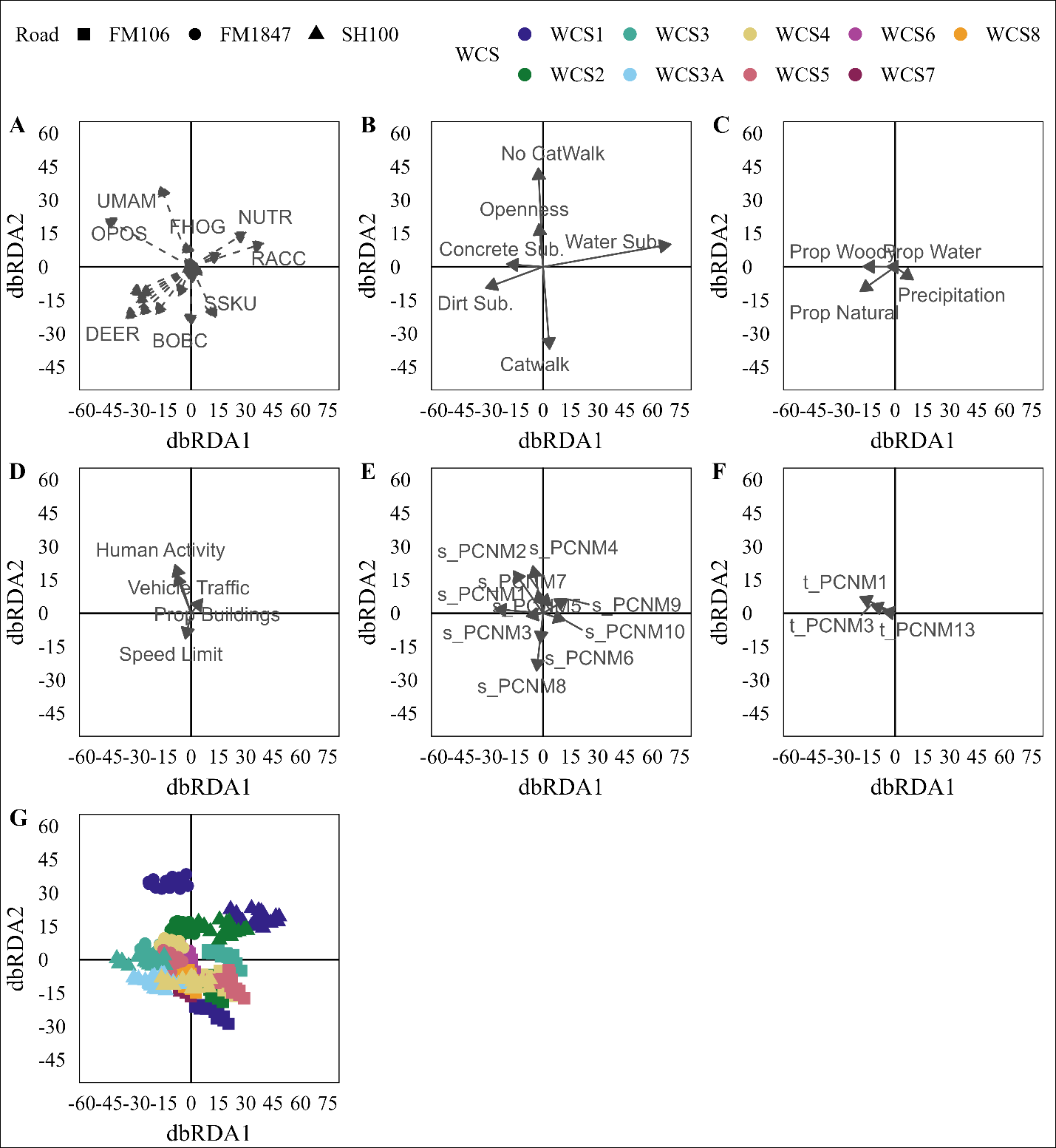


Figure S6.1: Bi-plot based on distance-based redundancy analysis (dbRDA) between dbRDA axes 1 and 2 showing the axis correlations with A) species scores, B) spatial, C) temporal, D) structural, E) environmental, and F) anthropogenic characteristics, and G) raw site scores for total detections. Full species names are provided in Appendix S2.


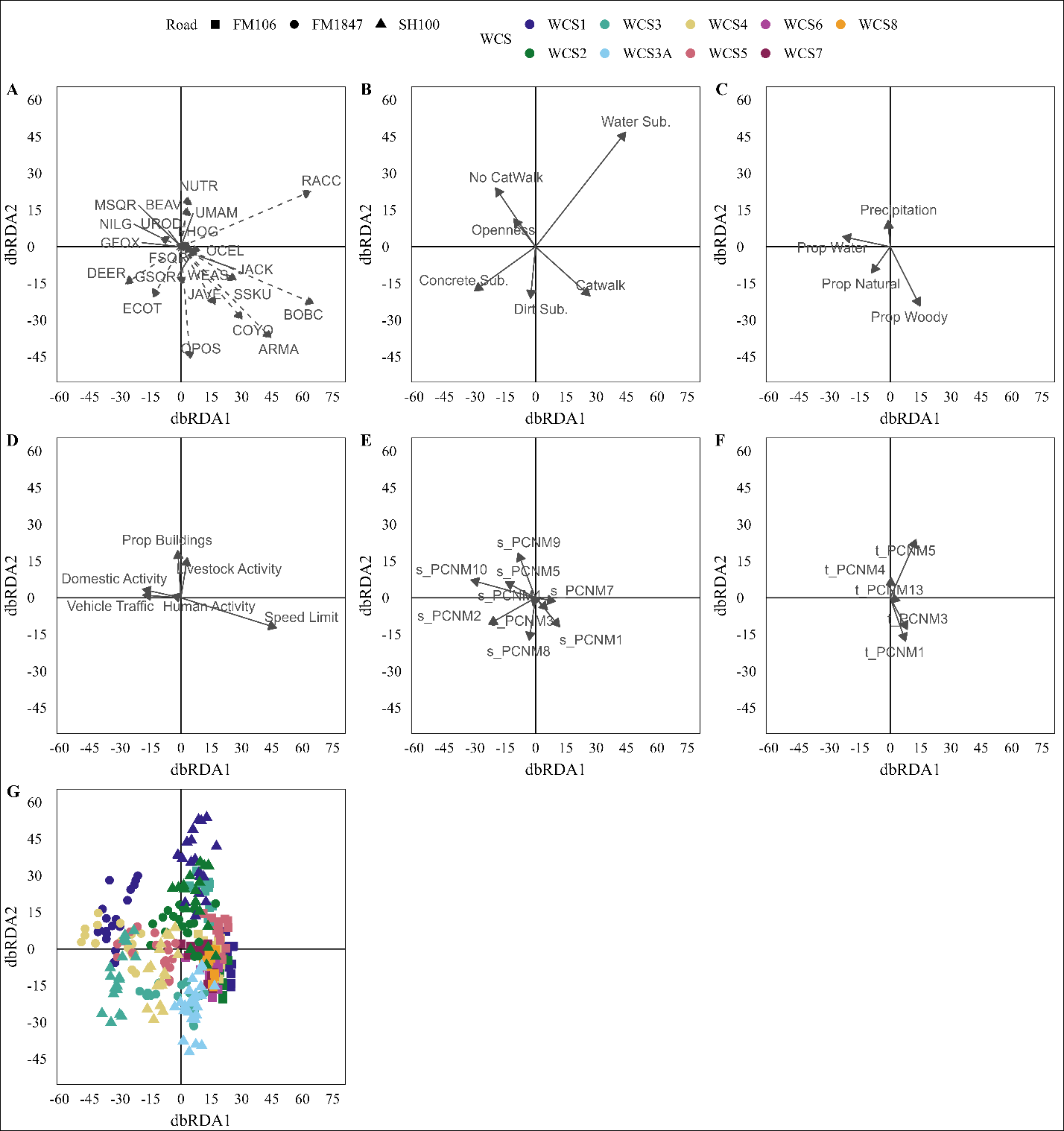


Figure S6.2: Bi-plot based on distance-based redundancy analysis (dbRDA) between dbRDA axes 1 and 2 showing the axis correlations with A) species scores, B) spatial, C) temporal, D) structural, E) environmental, and F) anthropogenic characteristics, and G) raw site scores for successful crossings. Full species names are provided in Appendix S2.


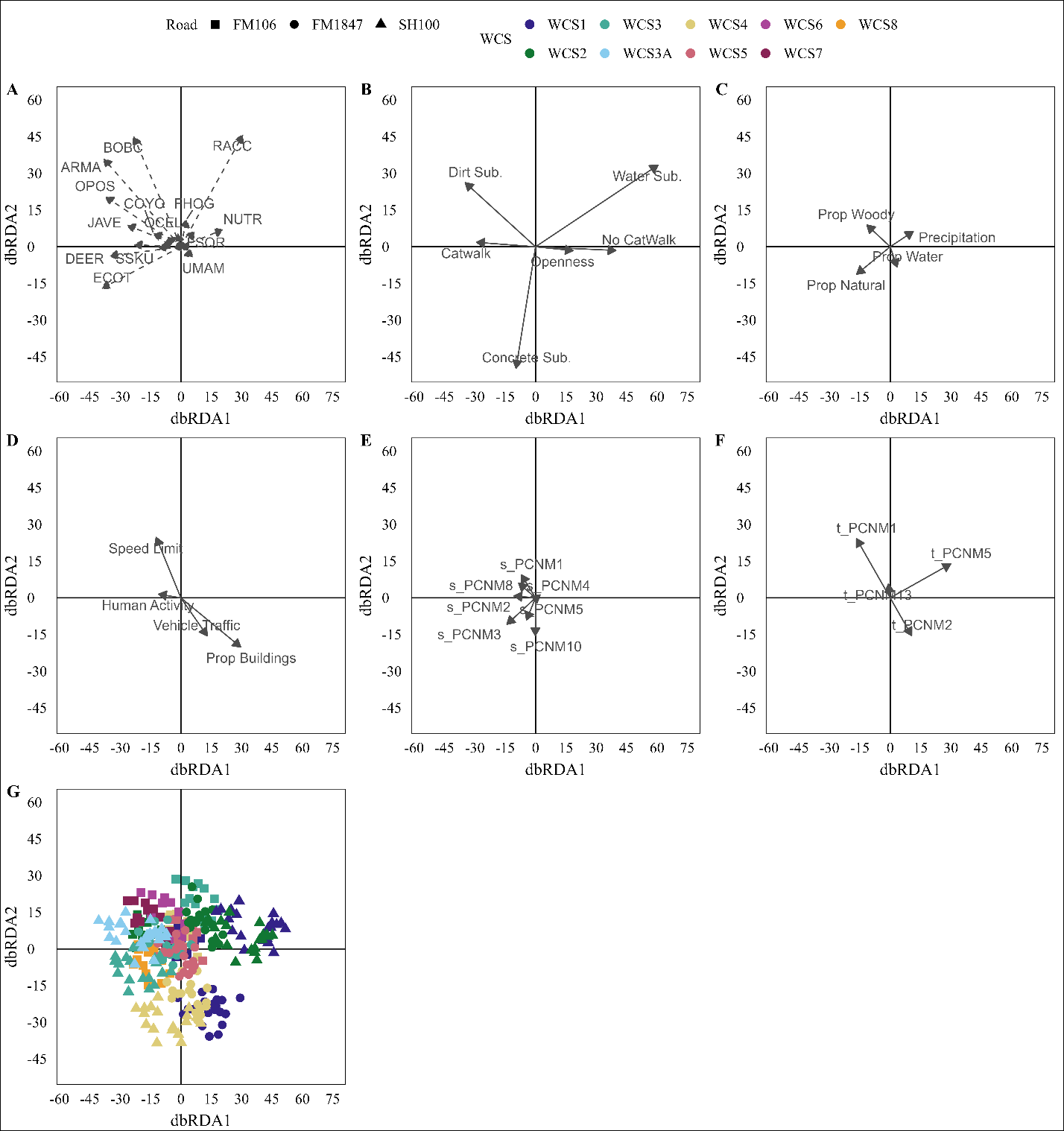


Figure S6.3: Bi-plot based on distance-based redundancy analysis (dbRDA) between dbRDA axes 1 and 2 showing the axis correlations with A) species scores, B) spatial, C) temporal, D) structural, E) environmental, and F) anthropogenic characteristics, and G) raw site scores for failed crossings. Full species names are provided in Appendix S2.

Figures S6.4-S6.7: ordination diagrams of the unfitted PCO axes, fitted PCO axes based on the full model, predicted PCO scores from the drop-one-site model, and the species scores for PCO axis 3. The PCO 3 axis was non-trivial only in the total detections and successful crossings analyses.


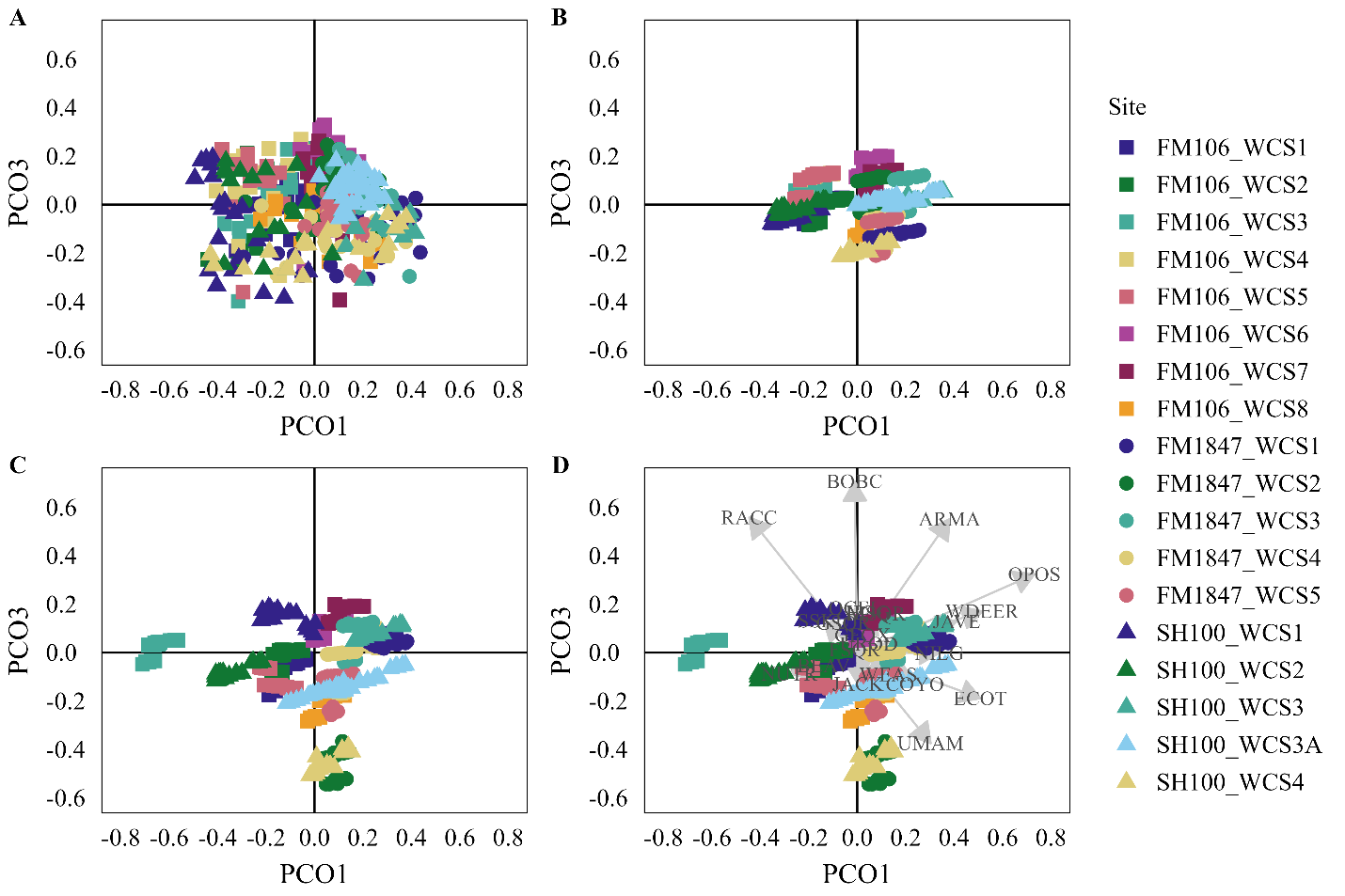


Figure S6.4: Ordination diagrams showing the A) raw principal coordinates scores (PCO), B) original fitted PCO scores (these can also be thought of the output of distance-based redundancy analysis), C) the predicted PCO scores from the models in which that site was dropped for PCO axes 1 and 3, and D) the species scores overlayed on the predicted PCO scores for the total detections analysis. Full species names are provided in Appendix S2.


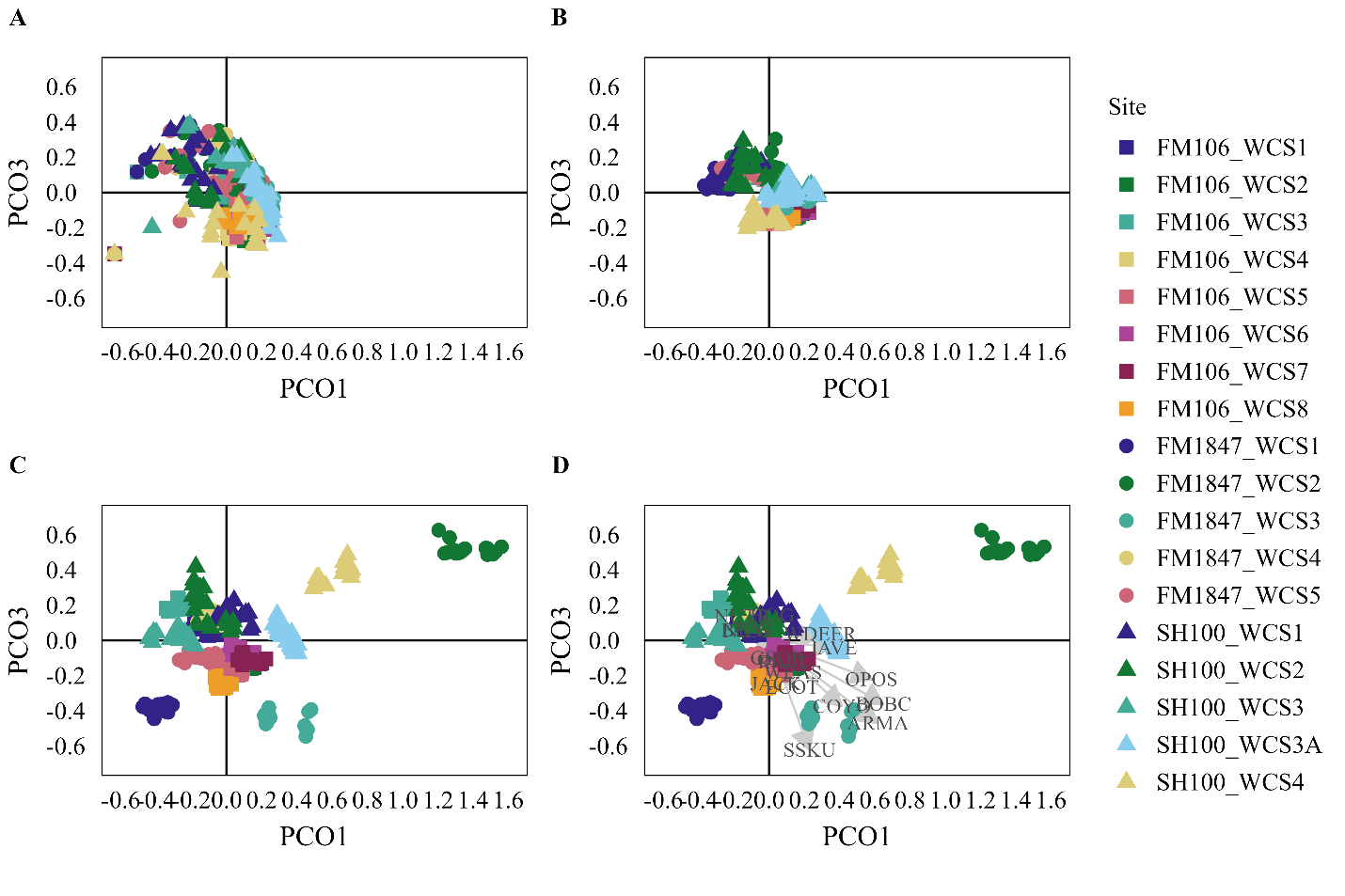


Figure S6.5: Ordination diagrams showing the A) raw principal coordinates scores (PCO), B) original fitted PCO scores (these can also be thought of the output of distance-based redundancy analysis), C) the predicted PCO scores from the models in which that site was dropped for PCO axes 1 and 3, and D) the species scores overlayed on the predicted PCO scores for the successful crossings analysis. Full species names are provided in Appendix S2.


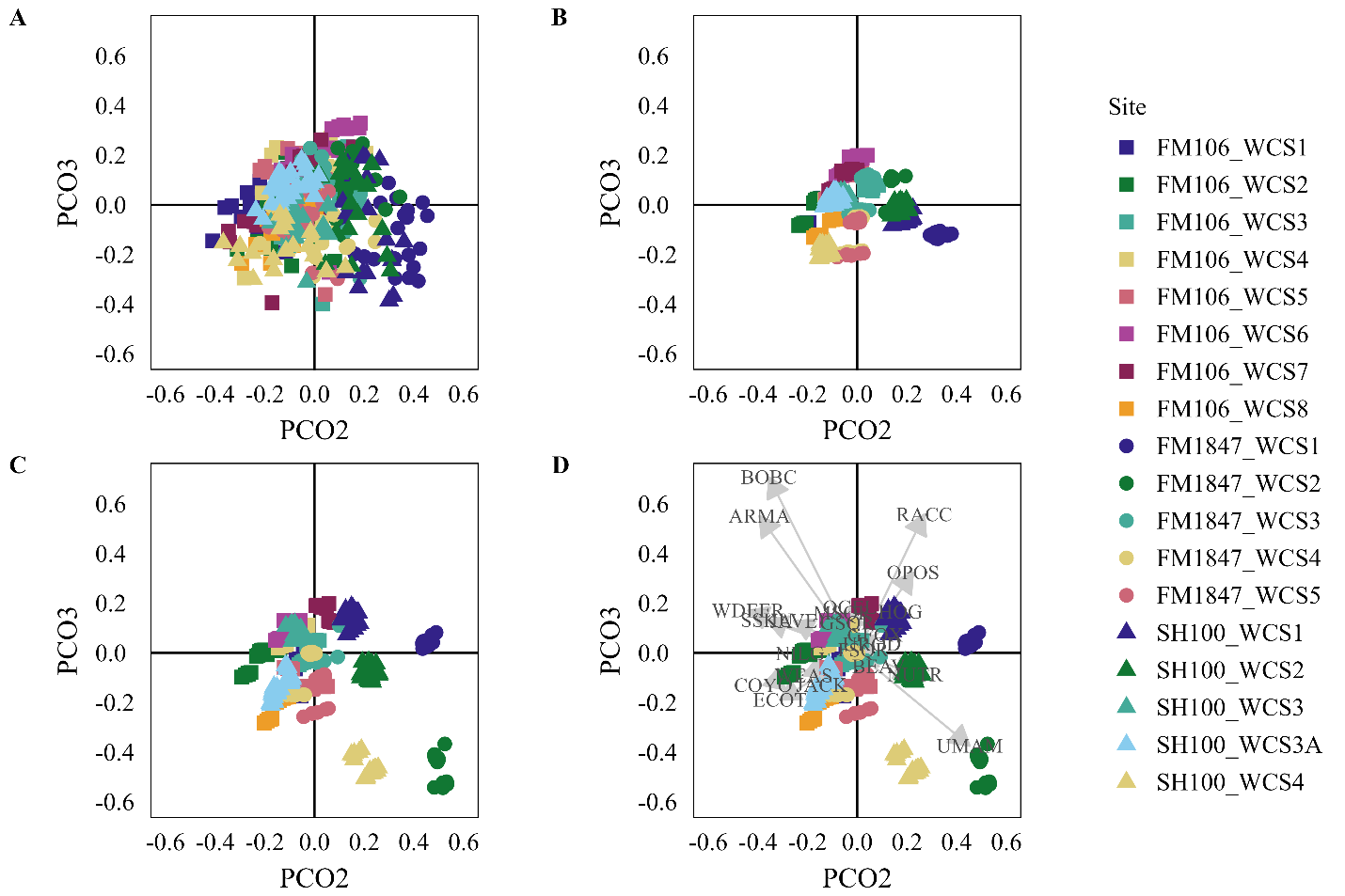


Figure S6.6: Ordination diagrams showing the A) raw principal coordinates scores (PCO), B) original fitted PCO scores (these can also be thought of the output of distance-based redundancy analysis), C) the predicted PCO scores from the models in which that site was dropped for PCO axes 2 and 3, and D) the species scores overlayed on the predicted PCO scores for the total detections analysis. Full species names are provided in Appendix S2.


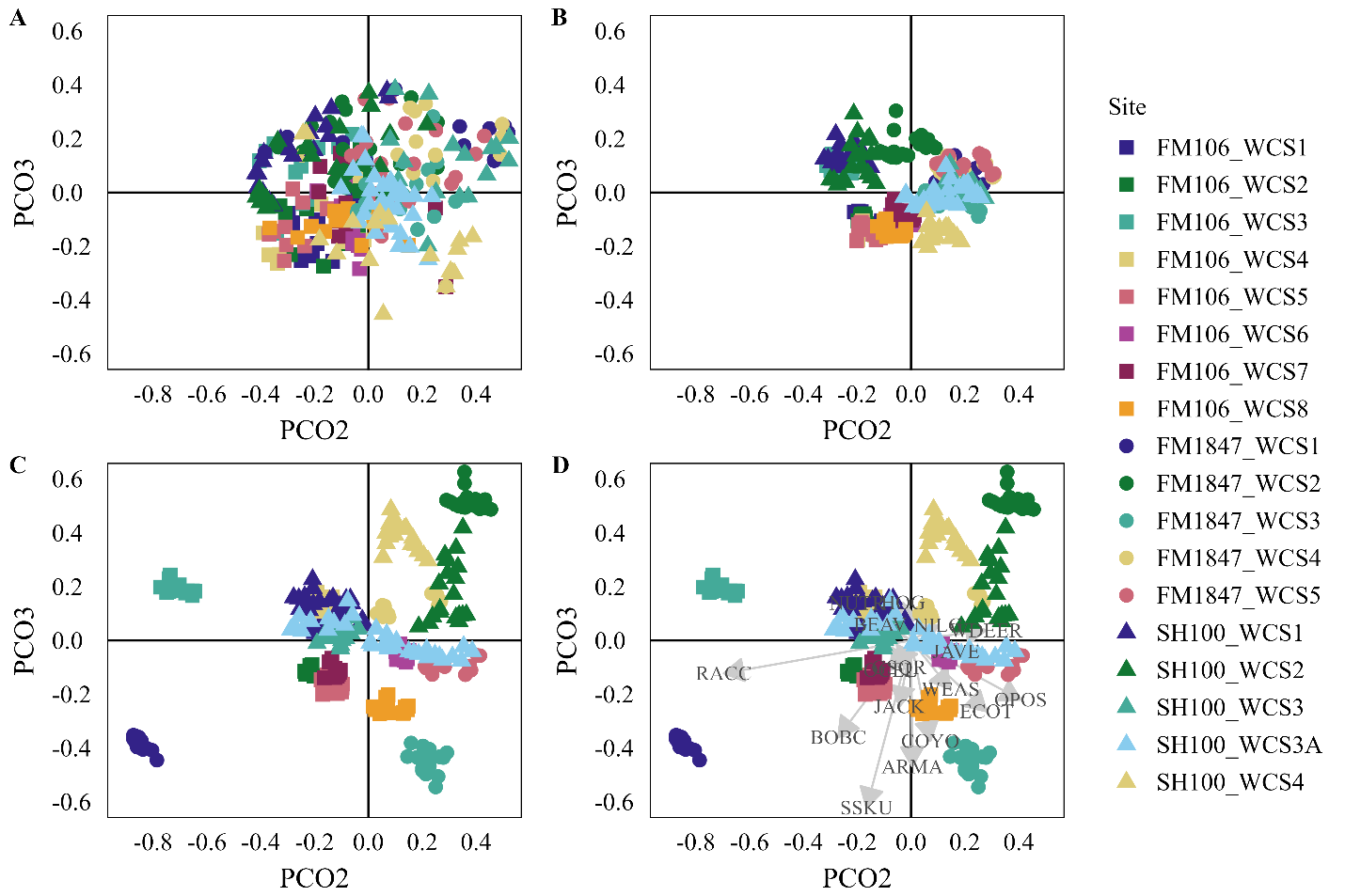


Figure S6.7: Ordination diagrams showing the A) raw principal coordinates scores (PCO), B) original fitted PCO scores (these can also be thought of the output of distance-based redundancy analysis), C) the predicted PCO scores from the models in which that site was dropped for PCO axes 2 and 3, and D) the species scores overlayed on the predicted PCO scores for the successful crossings analysis. Full species names are provided in Appendix S2.
