# Supplementary material for: Predicting species assemblages at wildlife crossing structures using multivariate regression of principal coordinates
Source: PLoS One. 2025 Oct 24;20(10):e0335193. doi: 10.1371/journal.pone.0335193 (PMC12551880; doi:10.1371/journal.pone.0335193)
Supplement: Attachment — Submitted filename: PlosOne_ResponseToReviewers_Rnd1_v2.docx [file pone.0335193.s008.docx]

**Journal and Editor Comments**

We have made all editorial changes required by the journal and updated our funding, acknowledgements, and data availability statements. We have moved our data repository to FigShare and provided DOIs for it and the R package used for data processing. Unfortunately we are unable to release the camera trap data because it contains ocelot locations, a federal endangered species. Our permit with US Fish and Wildlife Service and agreements with the Texas Department of Transportation prevents us from releasing any location information related to ocelots. We can still make this data available upon specific request. Figure 1 used imagery from the national agriculture imagery program and is in the public domain. The authorship issue was due to a missing “Jr.” in John Young’s name in the submission portal.

**Reviewer #1:**

Article is a bit not clear when you do not explain body size of mammals and their adaptive ecology. I suggest you re-write with addition of body size and nature of structures? I wonder have you mentioned about bio-engineering structures. It may some time attract or repel movement? Also elaborating drop-onsite method clearly would be good for readers.

We do mention that most of the WCSs were too small for large mammals such as deer or nilgai; however, both species used WCSs when they were large enough for them so we included them in the analyses. We believe that generally body size is not relevant for the purposes of this study where we were interested in the community composition of wildlife crossings, not in examining allometric relationships with crossing use. While this could be an interesting future avenue of study, in our study system, not all crossings in our system are large enough for all species, so it would be difficult to examine body size effects on crossing rates. As such, we have added the range of body size to our description of the species captured and added some additional discussion of deer and nilgai WCS use.

We are unclear what the reviewer means about bio-engineered wildlife crossings. While WCSs are often designed for particular species, this is often done based on assumptions about their behavior and preferences. Our study could help improve future WCS design by providing a method to test the potential efficacy of a WCS for a suite of species of interest and we explicitly state this in our discussion.

We have clarified that our drop-one-site method is similar to the leave-one-out-cross-validation technique for assessing model validity. In our case, the unit of observation is the WCS itself so our unit of observation for cross validation was all months in a WCS rather than a single sample (one month in one WCS). See below for further explanation of the method.

**Reviewer #2:**

1.     Line 35. Add a comma before "so" to properly separate the independent clauses.

We have added the requested comma.

2. The structure of Table 1 is excessively complex. There is too much information in this table. This table could be further broken down into two or more; e.g., information such as “timeline for WCS construction” could be a distinct table.

We have split Table 1 into two tables, one for the characteristics of the WCSs on the study roads and the other for the timeline of construction and monitoring of the WCSs.

3. Generally, predictive modeling should include both a training dataset and a testing dataset. This study did not adhere to this rule. Was a technique such as cross-validation, transfer learning, and leveraging a pretrained model used? A model without a separate test set could be difficult to assess for its true generalization ability and be misleading.

We agree that for predictive modeling, ideally we should have both a training dataset and a testing dataset and we acknowledge this in the methods, line 359. Because our sample size of WCSs was small (n = 18) and highly variable, we opted against leaving out a set of WCSs as a testing dataset. Instead we used a technique similar to LOOCV where we excluded all months from one WCS, reran the model, then predicted the dropped WCS using the fitted model. We originally described this in the paragraph starting on Line 363 and have clarified how our drop-one-site approach is similar to LOOCV.

4. Why do you not detrend the spatial and temporal autocorrelation in this study?

We detrended the spatial and temporal autocorrelation using a dbMEM when we ran our variation partitioning. However, for our predictive modeling, we wanted to create a model that could be used to predict future use of other (unbuilt) wildlife crossings in the system and be generalizable to other systems. The issue with using dbMEM to detrend autocorrelation is that the dbMEM axes that get included are model specific and you cannot add new values of dbMEM axes for additional testing data without changing the whole model. This makes the model useless for prediction purposes. We did, however, test the differences in model fit between a model fitted with raw coordinates and months and a detrended model and found little to no difference in the model fits, indicating that, for our purposes at least, detrending the autocorrelation was likely not necessary and results in a less useful predictive model. We have added some clarification to Lines 334-340.

5.     Line 503 “Our model performed relatively well for total detections but performed much worse for successful crossings and failed crossings.” A further explanation is needed regarding the poorer performance in both successful and failed crossings. This is a limitation that needs to be worked upon in the future study.

We believe that the poor performance of the successful crossings and failed crossings models was due to the additional factors that influence actual crossing rates, such as the presence of vehicles, noise or light levels, or the individual’s behavior/reason for being at the WCS itself. Another study in the same study area showed that opossums avoid crossing through WCSs when there is more vehicle noise [1]. Additionally, based on observation and videos in the study system, many species forage around WCSs and they may approach the WCS, appearing to interact with the structure on still image camera traps, as used in this study, but not actually have intentions of crossing.

**References**

1.	Yamashita TJ, Tanner AM, Tanner EP, Scognamillo DG, Tewes ME, Young Jr JH, et al. The importance of soundscapes in monitoring wildlife crossing structures. Ecological Applications. In Review.
